# Nonlinear kernel-based high-dimensional inference for set-based genetic association studies

**DOI:** 10.1093/bib/bbag275

**Published:** 2026-05-27

**Authors:** Zechen Zhang, Hui Yang, Meilin Zhu, Ran Guo, Fuzhao Chen, Hui Dong, Yuehua Cui, Haitao Yang

**Affiliations:** Division of Health Statistics, School of Public Health, Hebei Medical University, 361 East Zhongshan Road, Shijiazhuang, Hebei 050017, P.R. China; Hebei Key Laboratory of Environment and Human Health, School of Public Health, Hebei Medical University, 361 East Zhongshan Road, Shijiazhuang, Hebei 050017, P.R. China; Hebei Key Laboratory of Forensic Medicine, School of Forensic Medicine, Hebei Medical University, 361 East Zhongshan Road, Shijiazhuang, Hebei 050017, P.R. China; Division of Health Statistics, School of Public Health, Hebei Medical University, 361 East Zhongshan Road, Shijiazhuang, Hebei 050017, P.R. China; Hebei Key Laboratory of Environment and Human Health, School of Public Health, Hebei Medical University, 361 East Zhongshan Road, Shijiazhuang, Hebei 050017, P.R. China; Hebei Key Laboratory of Forensic Medicine, School of Forensic Medicine, Hebei Medical University, 361 East Zhongshan Road, Shijiazhuang, Hebei 050017, P.R. China; Division of Health Statistics, School of Public Health, Hebei Medical University, 361 East Zhongshan Road, Shijiazhuang, Hebei 050017, P.R. China; Division of Health Statistics, School of Public Health, Hebei Medical University, 361 East Zhongshan Road, Shijiazhuang, Hebei 050017, P.R. China; Division of Health Statistics, School of Public Health, Hebei Medical University, 361 East Zhongshan Road, Shijiazhuang, Hebei 050017, P.R. China; Department of Neurology, Second Hospital of Hebei Medical University, 215 West Heping Road, Shijiazhuang, Hebei 050000, P.R. China; Department of Statistics and Probability, Michigan State University, 619 Red Cedar Road, East Lansing, MI 48824, United States; Division of Health Statistics, School of Public Health, Hebei Medical University, 361 East Zhongshan Road, Shijiazhuang, Hebei 050017, P.R. China; Hebei Key Laboratory of Environment and Human Health, School of Public Health, Hebei Medical University, 361 East Zhongshan Road, Shijiazhuang, Hebei 050017, P.R. China; Hebei Key Laboratory of Forensic Medicine, School of Forensic Medicine, Hebei Medical University, 361 East Zhongshan Road, Shijiazhuang, Hebei 050017, P.R. China

**Keywords:** kernel method, nonlinear high-dimensional inference, *P*-value combination, omnibus test, SNP–set association

## Abstract

Nonlinear genetic architectures, including epistasis and threshold effects, are increasingly recognized as contributors to complex disease risk, yet most existing SNP-set association tests rely on linear modeling assumptions, resulting in reduced power and unstable inference when genetic effects are nonlinear or heterogeneously distributed across variants. To address this limitation, we propose a nonlinear high-dimensional inference framework for set-based genetic association analysis that integrates scalable kernel representations with valid statistical inference. The framework combines distance correlation-based sure independence screening to reduce ultra-high dimensional predictors, kernel principal component analysis with Nyström approximation for nonlinear feature extraction, and de-sparsified LASSO to enable asymptotically valid hypothesis testing in high dimensions, together with a two-stage omnibus testing strategy that adaptively aggregates evidence across complementary signal models. Extensive simulation studies demonstrate that the proposed method maintains well-calibrated Type I error and consistently achieves higher power than established set-based approaches, including Sequence Kernel Association Test and adaptive Sum of Powered Score test, particularly under nonlinear and heterogeneous genetic effect scenarios, while remaining competitive in linear settings. Application to Alzheimer’s Disease Neuroimaging Initiative data identifies gene-level associations with brain regional volumes that converge on neuronal excitability, calcium signaling, and cytoskeletal regulation, biological processes centrally implicated in neurodegeneration. Together, this work provides a robust and scalable framework for nonlinear set-based inference in genome-wide studies, expanding the analytical toolbox for dissecting complex genetic contributions to disease.

## Introduction

Genome-wide association studies (GWAS) have transformed our understanding of the genetic basis of complex traits, identifying thousands of disease-associated variants across diverse phenotypes [1]. Yet, the proportion of phenotypic variance explained by these discoveries remains modest, contributing to the longstanding “missing heritability” problem [2, 3]. Much of this gap arises because marginal single-SNP analyses lack sensitivity to weak, polygenic, and interacting effects and must operate under a severe multiple-testing burden [4–6].

Set-based association tests provide an important step forward by aggregating genetic information across biologically meaningful units, genes, pathways, and regulatory modules, thereby increasing power for dispersed effects and improving functional interpretability [7]. Representative methods, including burden tests [8], Sequence Kernel Association Test (SKAT) [9], gene-set enrichment approaches [10], and adaptive procedures such as aSPU [11], have become widely used tools for GWAS. However, the one-set-at-a-time nature limits their ability to account for cross-set dependence, co-regulation, or interaction structures, and makes it difficult to disentangle redundant signals across correlated sets [12]. Consequently, correlated sets may appear jointly significant due to overlapping biological processes, and conditional effects cannot be isolated [3, 6]. Penalized multi-set modeling frameworks [13–17] address some of these challenges by enabling simultaneous analysis across multiple gene sets, but they rarely provide valid statistical inference, such as *P*-values or confidence intervals, at a gene-set level. Although recent advances in high-dimensional inference offer de-biased estimators for valid uncertainty quantification [18, 19], such approaches remain inherently linear and cannot accommodate nonlinear genetic architectures.

Growing biological evidence indicates that nonlinear genetic effects, including epistasis, gene–environment interactions, and threshold-like dose–response patterns, play critical roles in many complex diseases [20]. These nonlinearities can distort inference, obscure mechanistic interpretation, and substantially reduce power if analyzed under linear assumptions. For example, the APOE-ε4 allele exhibits a nonlinear, dose-dependent impact on ad risk [21, 22]. Despite their importance, robust nonlinear inference remains difficult in high-dimensional set-based analyses due to challenges in representing nonlinear structure, selecting appropriate model complexity, and maintaining valid statistical guarantees.

To address these limitations, we introduce a nonlinear high-dimensional omnibus testing framework designed specifically for set-based genetic association analysis. Our approach provides three conceptual advances: it enables scalable nonlinear representation of SNP sets, supports valid uncertainty quantification in high dimensions, and adaptively integrates evidence across heterogeneous genetic architectures. Unlike existing linear or marginal methods, the framework is designed to remain robust under both cumulative weak and dominating strong signal structures, and to accommodate a wide spectrum of nonlinear genotype–phenotype relationships. Extensive simulations and analysis of the Alzheimer’s Disease Neuroimaging Initiative (ADNI) dataset demonstrate that this framework achieves well-calibrated Type I error, strong power under nonlinear models, and biologically coherent gene–phenotype associations. Together, these results establish a generalizable and biologically informed paradigm for nonlinear set-based inference in complex disease genomics.

### Statistical methods

**Figure 1:**
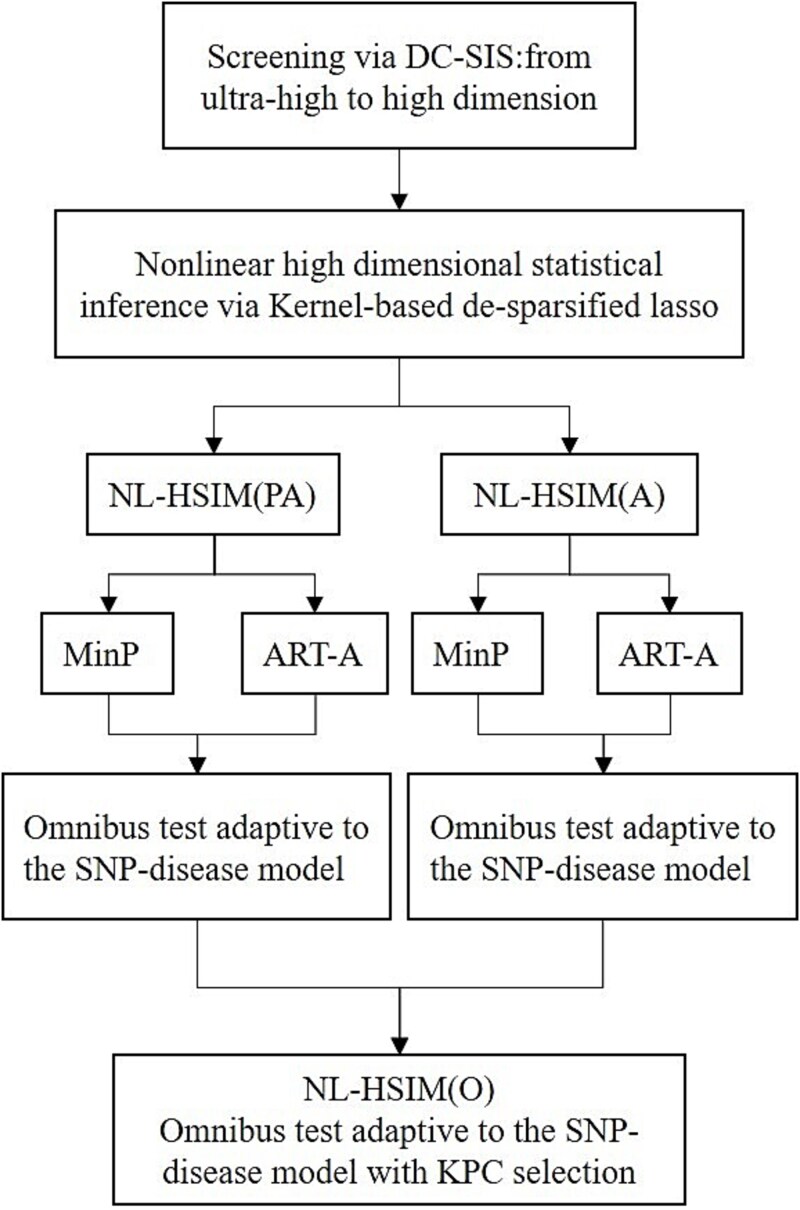
The flowchart of the nonlinear SNP-set association test leveraging the DSSM and CWSM hypotheses. NL-HSIM: Nonlinear high-dimensional inference model, PA: the strategy of extracting the KPC by combining pre-image reconstruction error and average eigenvalue A: the strategy of extracting KPCs using the average eigenvalue criterion alone MinP: the minimum *P*-value statistic ART-A: adaptive augmented rank truncated product O: Omnibus test jointly addressing adaptivity to the SNP–disease model and KPC selection.

In GWAS, the number of predictors often far exceeds the sample size, posing an ultra-high dimensional challenge. Moreover, the relationship between genetic variants and disease outcomes can be nonlinear, which limits the effectiveness of conventional linear models. To address both dimensionality and nonlinearity, we propose a two-stage nonlinear inference framework designed for high-dimensional association testing. In the first stage, we employ distance correlation-based sure independence screening (DC-SIS) [23] to efficiently reduce the data from ultra-high to high dimensionality. This model-free approach, built on distance correlation [24], captures both linear and nonlinear dependencies between predictors and the outcome, ensuring that informative features are retained. In the second stage, a kernel-based high-dimensional inference procedure is applied to the screened features to detect nonlinear associations. The overall workflow of our proposed framework is illustrated in Fig. 1, and the detailed procedures of the two-stage method are described below.

### Variable screening

Because genome-wide and other high-throughput studies operate in an ultra-high-dimensional setting, we first applied DC-SIS [23] to reduce the predictor space. This model-free procedure detects both linear and nonlinear dependence between predictors and the outcome, thereby retaining informative variables while substantially reducing the computational burden of downstream kernel analyses. In simulation studies, we retained the top *n*/l*og n* predictors, where *n* is the sample size. Detailed algorithmic steps for DC-SIS are provided in the Supplementary Materials [19, 25]. In the real-data analysis, DC-SIS was applied as an initial screening step over the candidate predictors, including both SNPs and covariates. Covariates, if retained, were carried forward only as linear adjustment variables and were not included in the kernelization step. Only the screened SNPs were mapped to genes and subjected to gene-wise kernel construction and KPCA. The resulting KPCs were then concatenated with the retained covariates for downstream de-sparsified LASSO inference.

### Nonlinear high-dimensional inference via kernel principal component analysis and de-sparsified LASSO

Following screening, KPCA was applied to each retained feature set to derive nonlinear low-dimensional representations in a reproducing kernel Hilbert space. The retained kernel principal components (KPCs) were subsequently used as predictors in a de-sparsified LASSO regression to perform valid high-dimensional inference. This procedure enables set-level hypothesis testing while accommodating nonlinear dependence among predictors.

### Kernel principal component analysis-based nonlinear component extraction

#### Kernel construction

To model nonlinear relationships among predictors within each set, we employed a Gaussian radial basis function kernel


(1) \begin{equation*} \mathbf{K}\left({x}_i, {x}_j\right)=\exp \left.\left(-\frac{{\left\Vert{x}_i-{x}_j\right\Vert}^2}{2{\sigma}^2}\right.\right) \end{equation*}

which induces an implicit infinite-dimensional feature mapping and provides a smooth, nonparametric similarity representation. Its locality property enables effective modeling of distance-dependent variation beyond linear covariance in high-dimensional biological data. The bandwidth parameter *σ* was determined in a data-driven manner as the square root of the average pairwise squared Euclidean distance [26] among samples within each set, ensuring comparable nonlinear scaling across feature sets. Because the proposed framework is intended primarily for statistical inference rather than prediction-oriented optimization, *σ* was not further tuned by cross-validation or grid search, as prediction-based criteria may not align with inferential objectives such as stability, error control, and power. We therefore regard this specification as a practical and reproducible default rather than a universally optimal choice.

#### Nyström approximation for scalable kernel principal component analysis

To ensure computational scalability, the full kernel matrix $\mathbf{K}$ was approximated using the Nyström method, yielding a low-rank representation, $\overset{\sim }{\mathbf{K}}=C{W}^{\dagger }{C}^T$where *C* denotes kernel evaluations between all samples and a subset of landmark points, and ${W}^{\dagger }$ is the Moore–Penrose pseudoinverse of the landmark submatrix. This approximation reduces computational complexity from *O*(*n*^3^) to *O*(*nm*^2^) (*m* ≪ *n*) while preserving the dominant spectral structure required for subsequent KPCA.

#### Retention of kernel principal components

To determine the number of KPCs to retain, we used two complementary selection strategies that capture distinct signal structures.

(i) Average eigenvalue criterion [27].

Following KPCA, the centered kernel matrix ${\overset{\sim }{\mathbf{K}}}_C\in{\mathbb{R}}^{n\times n}$ was eigen-decomposed as ${\overset{\sim }{\mathbf{K}}}_C{\nu}_k={\lambda}_k{\nu}_k$，${\lambda}_1\ge{\lambda}_2\ge \dots \ge{\lambda}_n\ge 0$, we computed the mean eigenvalue


(2) \begin{equation*} \overline{\lambda}=\frac{1}{n}\sum_{k=1}^n{\lambda}_k \end{equation*}

and retained KPCs with ${\lambda}_k>\overline{\lambda}$.

This criterion favors components that contribute above-average nonlinear variance and perform well under cumulative weak-signal models (CWSM), where informative structure is distributed across many moderate eigen-directions.

(ii) Pre-image–guided component selection with average eigenvalue refinement (PA)

To better accommodate scenarios in which a small number of dominant nonlinear directions explain most of the signal, as is typical in dominating strong-signal models (DSSM), we adopted a two-stage hybrid strategy.

**Stage 1: Pre-image reconstruction for coarse dimensionality selection [** **28** **[.**

Let $X\in{\mathbb{R}}^{n\times p}$denote the standardized predictor matrix, with rows *x_r_*(*r* = 1,…,*n*), and $\phi \left(\cdotp \right)$ the Gaussian feature map associated with the kernel. For a candidate number of components *h*, we perform 10-fold cross-validation. In fold *i*, the training indices *T_i_* are used to compute the centered kernel matrix and to run KPCA, retaining the first *h* KPCs. The corresponding truncated feature-space mean is


(3) \begin{equation*} {\overline{\phi}}_i^{(h)}=\frac{1}{\mid\! {T}_i\!\mid}\sum_{r\in{T}_i}\phi \left({x}_r\right) \end{equation*}

Its pre-image in the input space is defined as


(4) \begin{equation*} {\hat{x}}_i^{(h)}=\arg{\min}_x{\left\Vert \phi (x)-{\overline{\phi}}_i^{(h)}\right\Vert}_{\mathcal{H}}^2 \end{equation*}

which we compute using Mika’s fixed-point algorithm for Gaussian kernels. Let


(5) \begin{equation*} {\mu}_i=\frac{1}{\mid{S}_i\mid}\sum_{r\in{S}_i}{x}_r \end{equation*}

denote the empirical mean of the held-out samples in fold *i* (with test indices *S_i_*). The fold-wise reconstruction error for dimension *h* is then


(6) \begin{equation*} R{E}_i(h)={\left\Vert{\mu}_i-{\hat{x}}_i^{(h)}\right\Vert}_2^2 \end{equation*}

and the 10-fold reconstruction error


(7) \begin{equation*} RE(h)=\frac{1}{10}\sum_{i=1}^{10}R{E}_i(h) \end{equation*}

measures how well the truncated feature space with *h* components preserves nonlinear structure in the original predictors. We identify


(8) \begin{equation*} {h}_{\mathrm{pre}}=\arg{\min}_h RE(h) \end{equation*}

as a coarse upper bound on the number of KPCs needed to capture dominant nonlinear signals.

Detailed algorithmic steps and implementation details for the pre-image reconstruction and cross-validation procedure are provided in the Supplementary Methods, and a pseudocode summary is provided in Supplementary Table S1.

**Stage 2: Average eigenvalue refinement.**

The pre-image stage provides an upper bound *h*_pre_. Within the top-ranked *h*_pre_ components, we further apply the average eigenvalue rule to retain only components with eigenvalues exceeding the mean.

This refinement step prevents the pre-image criterion from selecting overly large dimensions influenced by noise or kernel over-smoothing.

Thus, the PA procedure yields a two-step selection: Pre-image selects a signal-preserving range (good for strong signals); Average eigenvalue trims within-range noise components (improves stability and parsimony).

Detailed algorithmic steps, fold assignments, and convergence settings for the pre-image computation are provided in the Supplementary Methods.

### De-sparsified LASSO inference on kernel representations

After kernel construction and KPCA, the retained KPCs, together with the covariates carried forward separately from the screening stage, were incorporated into a de-sparsified LASSO model for valid statistical inference in the high-dimensional setting [18]. It is important to note that the theoretical requirements for de-sparsified LASSO, such as the compatibility condition, are imposed on the design matrix formed by these KPCs rather than the post-screening raw SNP matrix.

Unlike standard LASSO, which yields biased estimates due to ℓ₁ penalization, the de-sparsified formulation adds a bias-correction term derived from nodewise regressions, producing asymptotically normal estimators for each KPC. Because the KPCA procedure effectively compresses redundant information and truncates directions corresponding to near-zero eigenvalues, it alleviates the instability caused by the strong collinearity typically found in genomic data.

This helps ensure that the final KPC-level design matrix maintains the necessary regularity for valid inference. This framework enables the construction of confidence intervals and valid *P*-values that quantify the strength of association between nonlinear kernel features and the outcome. The resulting *P*-values are aggregated at the feature-set (e.g. gene) level in the following omnibus-testing stage. Algorithmic and estimation details are given in the Supplementary file.

### Adaptive omnibus testing for model and component selection uncertainty

To integrate evidence across multiple SNP-disease models and the selection of the number of KPC strategies, we design a two-stage omnibus test (NL-HSIM(O)) that operates on the de-sparsified LASSO outputs.

In **Stage 1**, within each KPC retention strategy (PA or A), *P*-values from de-sparsified LASSO are combined using MinP (which is sensitive to sparse, strong effects) and ART-A (which is effective for dense, weak signals). These two complementary set-level statistics are then merged through the Cauchy combination test [29], yielding a model-adaptive *P*-value, i.e. robust to unknown underlying effect structures (CWSM versus DSSM).

In **Stage 2**, to address uncertainty in KPC retention itself, the omnibus *P*-values from NL-HSIM(PA) and NL-HSIM(A) are further combined using another Cauchy aggregation, forming a unified omnibus *P*-value for each set. This hierarchical integration simultaneously adapts to the underlying SNP–disease model (sparse vs. dense signals) and to the KPC retention strategy (PA vs. A), thereby enhancing robustness to both model misspecification and dimensional uncertainty in kernel representations. Comprehensive derivations, implementation details, and additional validation results for the omnibus procedure are provided in the Supplementary file.

### Validation of the proposed method with simulation studies

We conducted extensive simulations to evaluate the performance of the proposed framework across diverse scenarios. The simulation design followed the protocol of Morris [30], widely used in genomic studies. To reflect real-world data structures, we separately simulated continuous predictors (e.g. gene expression, methylation) and discrete predictors (e.g. SNPs). The outcome variable Y was continuous, aligning with typical quantitative traits in omics research. This design allows direct assessment of the method’s capacity to model complex associations between high-dimensional features and continuous phenotypes.

#### Aims

The primary aim of our simulation study is to evaluate the control of Type I error under the null hypothesis (i.e. no association between predictors and phenotype) and to assess the statistical power of our proposed framework under various conditions. To specifically examine the advantage of NL-HSIM in capturing nonlinear effects, we included simulation settings with nonlinear relationships between predictors and the outcome. Simulation scenarios varied in sample size, predictor types (discrete and continuous), within-group correlation, and genetic-disease models. Case I was designed primarily as a simplified discrete-predictor setting to isolate the gain from nonlinear representation learning relative to its linear counterpart. Additional comparisons with SKAT and aSPU for Case I are provided in Supplementary Tables S2–S6. By contrast, Case II serves as the main benchmarking scenario under more realistic chromosome-wide SNP dependence structures. Thus, Case I is intended mainly as a mechanism-oriented validation setting rather than the primary external benchmarking scenario. In all simulation figures, each plotted value represents an empirical Type I error rate or power estimate based on 1000 independent simulation replicates.

For comparison, we constructed a Linear-HSIM model by replacing the KPCA module in NL-HSIM with PCA, retaining principal components that explain over 85% of the cumulative variance. In addition, we included two widely used set-based association methods: the SKAT [9] and the aSPU [11], both of which are designed for large-scale gene-level association testing. These methods serve as robust baselines for assessing the power and robustness of our proposed approach across different genetic effect architectures.

#### Case I: Simulation for the small-scale discrete predictors

To evaluate model performance in gene-level inference, we simulated two groups of discrete predictors differing in dimension (e.g. genes with few vs. many SNPs) and the full data-generating procedures are provided in the supplement.

#### Case II: Simulation with the large-scale SNP data

We evaluated the statistical power of the proposed NL-HSIM(O) model under high-dimensional nonlinear settings and performed systematic comparisons with the established SKAT [9] and aSPU [11] methods. Detailed descriptions of the simulation design are provided in the Supplementary Materials.

#### Case III: Simulation with quantitative predictors

The proposed framework is not restricted to discrete SNP data and can be readily applied to quantitative predictors. For example, it can be used in pathway-based association studies where gene expression levels serve as predictors. Details of the simulation design are provided in the supplemental file due to space constraints.

### Validation using real data from the Alzheimer’s disease neuroimaging initiative study

To validate our proposed method, we analyzed data from the ADNI (https://adni.loni.usc.edu/), a longitudinal study launched in 2003 to investigate imaging, biomarker, and clinical indicators of Alzheimer’s disease (ad) progression. We focused on identifying genetic variants associated with the volumes of five brain regions: ventricles, hippocampus, entorhinal cortex, fusiform gyrus, and middle temporal gyrus.

### Data preprocessing

Quality control (QC) was performed on both SNPs and individuals. SNPs were excluded if they had a call rate < 95%, Hardy–Weinberg equilibrium *P*-value <1e-6, minor allele frequency < 1%, or missing rate > 5%. Individuals were removed if their genotype missing rate exceeded 10%, sex was inconsistent, or they carried X-chromosome variants. Genotype imputation was conducted using PLINK v1.9, and additional filters excluded samples with abnormal heterozygosity or cryptic relatedness. All SNPs were mapped to the GRCh37 reference genome. Further QC measures included removing individuals with heterozygosity exceeding three standard deviations or those with familial relationships.

After QC, the dataset included 299 763 SNPs mapped to 17 477 genes across 1043 individuals, spanning cognitively normal (CN), mild cognitive impairment, and ad diagnoses. Four covariates—age, sex, education, and APOE4 allele count—were included in the analysis (details in Supplementary Table S16).

## Results

### Results of simulation studies

We began by performing extensive simulation studies to assess the proposed method’s performance under varying conditions. Specifically, three distinct scenarios were considered: (i) a setting with a small number of discrete predictors, (ii) a large-scale GWAS context involving high-dimensional SNP data, and (iii) a case focusing on continuous quantitative predictors. Detailed specifications of each scenario can be found in the Statistical Methods section.

### Results of Case I: Simulation for the small-scale discrete predictors

**Figure 2:**
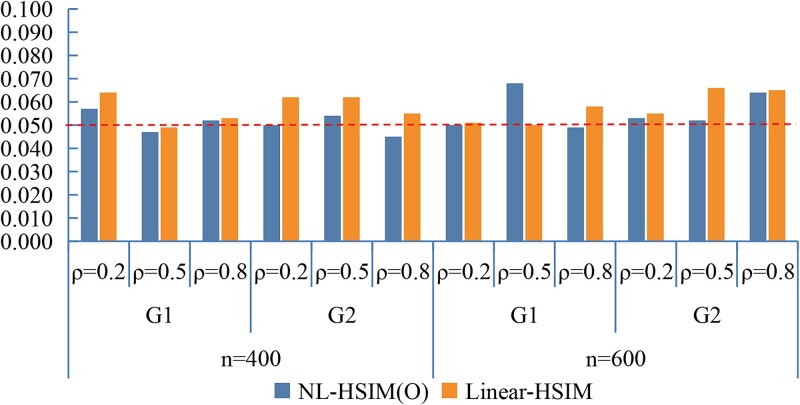
Empirical Type I error rates of NL-HSIM(O) and linear-HSIM under small-scale discrete predictor settings.

Figure 2 compares the Type I error rates between NL-HSIM(O) and Linear-HSIM under small-scale discrete predictor settings. Across both groups (G1 and G2), different sample sizes, and varying within-group correlations, NL-HSIM(O) maintained Type I error rates effectively at the nominal level.

**Figure 3:**
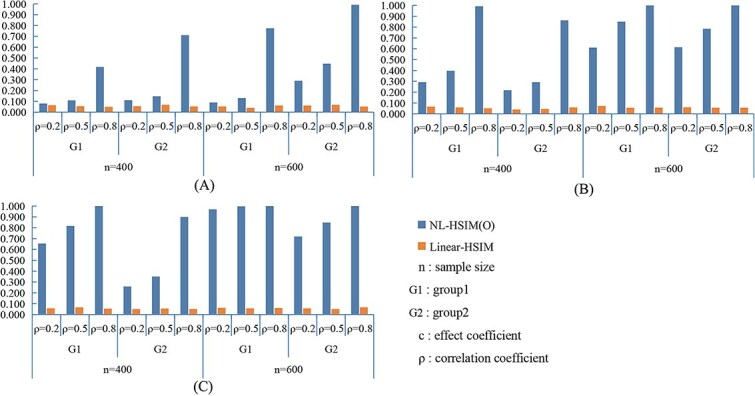
Comparison of NL-HSIM(O) and linear-HSIM in nonlinear CWSM simulation scenarios: (A) *c*_1_ = 0.1, *c*_2_ = 0.2; (B) *c*_1_ = 0.2, *c*_2_ = 0.4; (C) *c*_1_ = 0.3, *c*_2_ = 0.6.

**Figure 4:**
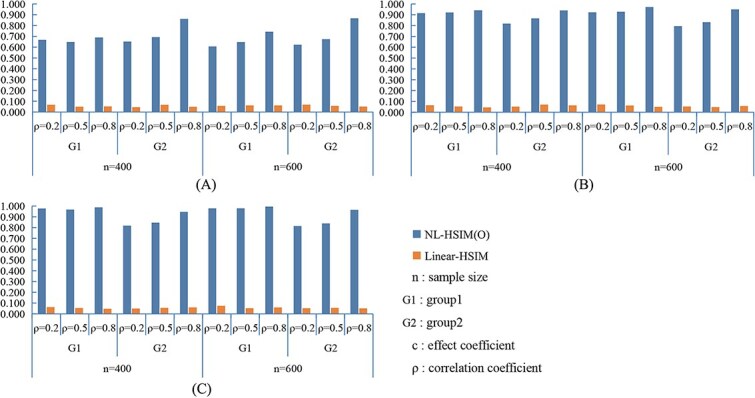
Comparison of NL-HSIM(O) and linear-HSIM in nonlinear DSSM simulation scenarios: (A) *c*_1_ = 0.5, *c*_2_ = 1; (B) *c*_1_ = 1, c_2_ = 3; (C) *c*_1_ = 1.5, *c*_2_ = 5.

Figures 3 and 4 present the statistical power results under nonlinear settings. In both nonlinear CWSM (Fig. 3) and nonlinear DSSM (Fig. 4) models, NL-HSIM(O) consistently outperformed Linear-HSIM. The performance gap widened as signal strength increased, indicating that NL-HSIM(O) better captures nonlinear genetic effects. For comparison, results from linear CWSM and linear DSSM scenarios are provided in Supplementary Figs S1 and S2, which show that both methods achieved comparable power when the underlying relationship was linear. Additional empirical Type I error results are shown in Supplementary Fig. S3. Additional power comparisons are shown in Supplementary Fig. S4, Fig. S5, Fig. S6, and Fig. S7.

Detailed numerical comparisons with SKAT and aSPU for Case I are provided in Supplementary Tables S2, Table S3, Table S4, Table S5, and Table S6**.**

### Results of Case II: Simulation using SNP data from chromosome 8

**Figure 5:**
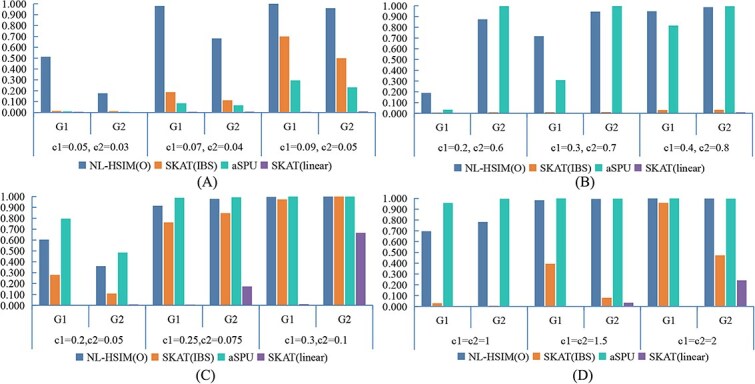
Power comparison of NL-HSIM(O), SKAT(IBS), aSPU, SKAT(linear) under chromosome-wide simulation scenarios with *n* = 600: (A) nonlinear CWSM; (B) nonlinear DSSM; (C) linear CWSM; (D) linear DSSM.

Figure 5 compares the empirical power of NL-HSIM(O), Linear-HSIM, SKAT, and aSPU across four chromosome-wide simulation scenarios (nonlinear/linear × CWSM/DSSM) using real SNP data from chromosome 8 of the ADNI cohort.

NL-HSIM(O) consistently achieved the highest or near-highest power in all configurations, reflecting its adaptability to both nonlinear and linear relationships and to varying signal distributions.

Among the SKAT variants, SKAT(IBS) generally outperformed SKAT(L), but both showed limited sensitivity to complex or weakly distributed signals, resulting in lower overall power than NL-HSIM(O). The aSPU test performed well when a few strong effects dominated and in some cases approached NL-HSIM(O)'s power; however, its performance declined sharply when effects were weaker or dispersed across multiple genes. While SKAT and aSPU each had strengths in specific scenarios, neither maintained high power across all conditions.

By contrast, NL-HSIM(O) delivered consistently strong and balanced detection, making it a robust and versatile tool for real-world genomic studies where the true genetic effect architecture is unknown. Additional comparisons are shown in Supplementary Fig. S8 and Fig. S9. Detailed numerical results for Case II are provided in Supplementary Table S7, Table S8, Table S9, and Table S10.

### Results of Case III: Simulation with quantitative predictors

**Figure 6:**
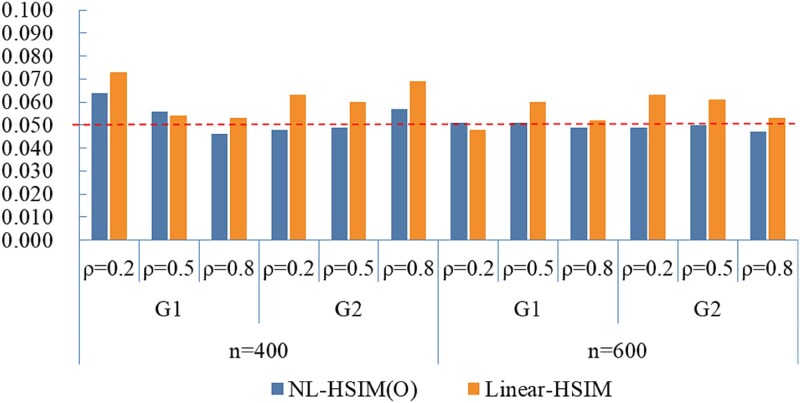
Empirical Type I error rates of NL-HSIM(O) and linear-HSIM under Case III simulations with continuous predictors.

**Figure 7:**
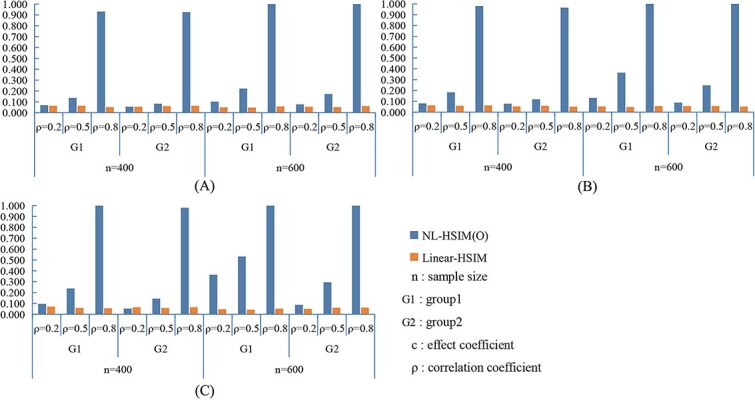
Power comparison of NL-HSIM(O) and linear-HSIM under nonlinear CWSM with continuous predictors: (A) *c*_1_ = 0.06, *c*_2_ = 0.07; (B) *c*_1_ = 0.07, *c*_2_ = 0.08; (C) *c*_1_ = 0.08, *c*_2_ = 0.09.

**Figure 8:**
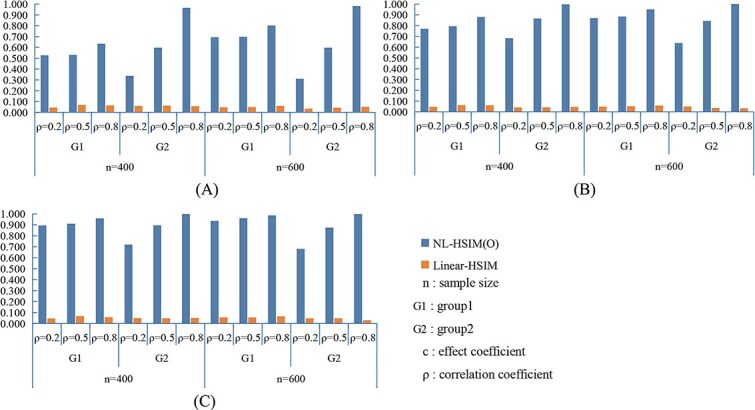
Power comparison of NL-HSIM(O) and linear-HSIM under nonlinear DSSM with continuous predictors: (A) *c*_1_ = 0.3, *c*_2_ = 0.4; (B) *c*_1_ = 0.4, *c*_2_ = 1.2; (C) *c*_1_ = 0.5, *c*_2_ = 2.

NL-HSIM(O) showed satisfactory control of the Type I error across all considered scenarios. Increasing the sample size from 400 to 600 led to more stable error control (Fig. 6). In nonlinear settings (Figs 7 and 8), NL-HSIM(O) generally exhibited higher power than Linear-HSIM, while in linear settings its performance was comparable. Additional power comparisons under linear settings are shown in Supplementary Fig. S10 and Fig. S11. Additional empirical Type I error and power comparisons among NL-HSIM(P), NL-HSIM(A), and NL-HSIM(PA) are shown in Supplementary Fig. S12, Fig. S13, Fig. S14, Fig. S15, and Fig. S16. Detailed numerical results for Case III are provided in Supplementary Table S11, Table S12, Table S13, Table S14, and Table S15.

### Results from real data analyses

We applied our proposed method to the ADNI dataset (https://adni.loni.usc.edu/)^1^, focusing on five brain region volumes as the response variables to demonstrate the method’s applicability. To account for potential nonlinear associations and reduce computational burden, we first performed distance correlation-based screening, selecting the top 10 000 most relevant predictors, including both covariates and SNPs based on DC-SIS. Detailed information of five brain regions after DC-SIS procedure is shown in Table 1.

**Table 1:** Numbers of retained predictors, mapped genes, and significant covariates for each brain region after DC-SIS screening.

**Dependent variables**	**Gene number**	**Covariates number**	**Covariates**
Ventricles	3115	2	Age, gender
Hippocampus	3286	3	Age, apoe4, gender
Entorhinal cortex	3228	4	Age, apoe4, educate, gender
Fusiform gyrus	3257	3	Age, educate, gender
Middle temporal gyrus	3187	3	Age, gender

**Figure 9:**
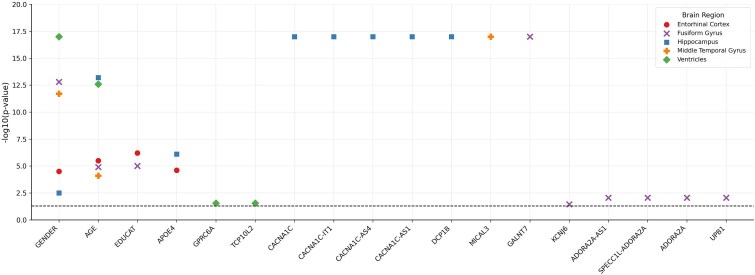
Significant volume-related associations with covariates and genes identified solely in five brain regions: ventricles, fusiform, middle temporal, hippocampus, and entorhinal cortex.

These candidate SNPs were subsequently mapped to their corresponding genes, and KPCs were extracted to summarize the genetic variation within each gene. Using a de-sparsified LASSO approach, we calculated *P*-values for both the KPCs and covariates, enabling gene-level inference. To further assess gene significance, we applied two complementary set-based methods, MinP and ART-A, and an omnibus test combining them via the Cauchy combination test. Multiple testing correction was then performed using the false discovery rate (FDR) at the gene level. A summary of the analysis results is presented in Fig. 9. Detailed real-data results are provided in Supplementary Table S17.

**Figure 10:**
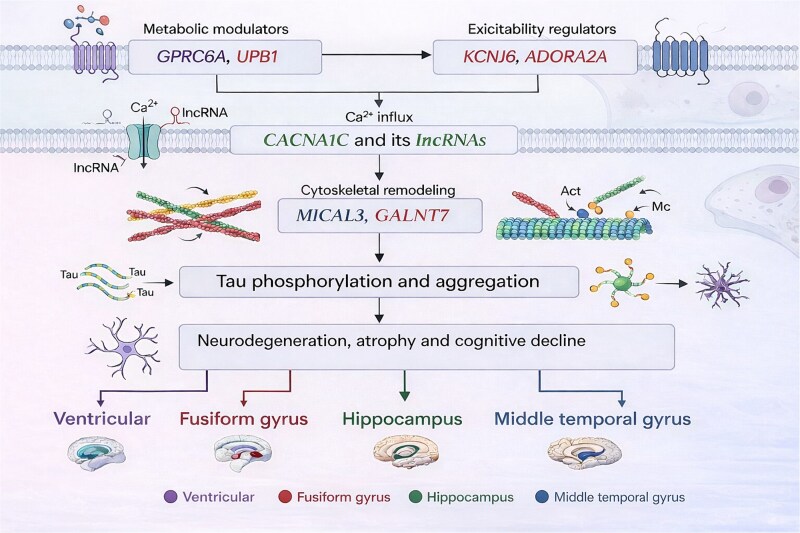
Proposed schematic biological interpretation of region-specific gene–brain region associations identified by NL-HSIM(O). Schematic summary of gene-level associations identified from the ADNI analysis using the proposed nonlinear set-based inference framework. Gene names are color-coded according to their associated brain regions, as indicated in the legend. The figure organizes the identified associations into literature-informed functional themes, including metabolic modulation (*GPRC6A*, *UPB1*), neuronal excitability regulation (*KCNJ6*, *ADORA2A*), calcium influx related to *CACNA1C* and its associated long non-coding RNAs, and cytoskeletal remodeling involving *MICAL3* and *GALNT7*. These connections are presented as a biologically motivated interpretive framework for understanding region-specific associations, rather than as direct mechanistic outputs of the statistical model.

Figure 9 displays the statistical significance of selected variables across five brain regions: Ventricles, Fusiform gyrus, Entorhinal Cortex, Hippocampus, and Middle Temporal gyrus.

A dashed horizontal line marks the threshold for statistical significance at FDR-adjusted *P*-value = .05, corresponding to $-{\log}_{10}(0.05)$  $\approx\, 1.3$.

### Covariate effects

Age and sex were significantly associated with the volumes of all five examined brain regions—entorhinal cortex, hippocampus, ventricles, fusiform gyrus, and middle temporal gyrus. In the fusiform gyrus and middle temporal gyrus, sex showed stronger associations than age, consistent with UK Biobank findings demonstrating more pronounced sex differences than age effects in temporal lobe morphology [31] and meta-analytic evidence of consistently larger temporal lobe volumes in males. In contrast, in the hippocampus, age demonstrated stronger associations than sex, consistent with longitudinal studies showing age-related hippocampal and medial temporal lobe atrophy in healthy aging and ad [32, 33].

APOE4 status was significantly associated with volumes of the entorhinal cortex and hippocampus, in line with evidence that APOE4 carriers experience accelerated atrophy in medial temporal lobe structures during preclinical and early ad [34, 35]. Higher educational attainment was associated with preserved entorhinal cortex volume, supporting the cognitive reserve hypothesis [36, 37].

### Gene effects

In the entorhinal cortex, no genes passed the FDR threshold, though strong covariate associations reinforce its role as an early locus of tau pathology.

In the ventricles, *GPRC6A* and *TCP10L2* were significant. *GPRC6A* is an amino acid–sensing G-protein–coupled receptor implicated in nutrient–*mTORC1* signaling and tau phosphorylation [38], whereas *TCP10L2* is a novel candidate gene with uncharacterized effects on brain morphology.

In the fusiform gyrus, significant genes included *KCNJ6*, *ADORA2A*, *ADORA2A-AS1*, *SPECC1L-ADORA2A*, *UPB1*, and *GALNT7.* These genes converge on excitability regulation, adenosine signaling, glycosylation, and metabolic pathways, all potentially modulating tau pathology [39–43].

In the hippocampus, *CACNA1C* and its associated lncRNAs (*CACNA1C-IT1*, *CACNA1C-AS4*, *CACNA1C-AS1*), together with *DCP1B*, were significant. *CACNA1C* encodes the Cav1.2 L-type calcium channel α₁C subunit, central to Ca^2+^-mediated tau phosphorylation [44], while *DCP1B* may influence tau pathology indirectly through mRNA turnover.

In the middle temporal gyrus, *MICAL3* was significant; this redox enzyme regulates cytoskeletal remodeling and has been linked to tau-mediated microtubule destabilization [45].

## Conclusion and discussion

Genetic association studies increasingly face the combined challenges of ultrahigh dimensionality and nonlinear effect structures, particularly in complex diseases such as ad, where highly polygenic, sparse, and potentially nonlinear effects may obscure genotype–phenotype relationships [1, 2, 46]. To address this setting, we developed NL-HSIM(O), a novel nonlinear high-dimensional inference framework that integrates KPCA, de-sparsified LASSO, and Nyström approximation for scalable set-based testing.

A key methodological issue in KPCA-based inference is the selection of retained KPCs. While the average eigenvalue rule remains useful, cumulative variance thresholds are often less suitable for KPCA because smooth kernels such as the RBF kernel typically produce long-tailed eigenspectra [47, 48]. In such settings, variance-threshold selection may retain many weak components, inflating downstream variance and reducing power [49–51]. Consistent with this, our simulations showed that variance-based selection was generally less effective than average-eigenvalue-based strategies. We therefore combined two complementary KPC selection approaches, PA and A, through a Cauchy combination test to improve robustness across heterogeneous genetic architectures.

Applied to the ADNI dataset, NL-HSIM(O) identified significant covariate and gene associations with the volumes of five brain regions: entorhinal cortex, hippocampus, ventricles, fusiform gyrus, and middle temporal gyrus. Age and sex were significantly associated with all five regions, with age showing stronger relevance to hippocampal atrophy and sex showing relatively stronger associations in the fusiform and middle temporal gyri [31–33, 52]. Educational attainment was associated with entorhinal cortex and fusiform gyrus volumes, consistent with cognitive reserve models [36, 37]. At the gene level, the framework recovered several loci with prior links to ad or neurodegeneration, including APOE4 [21, 53], *CACNA1C* [54], *ADORA2A* [55], and *GPRC6A* [56], while also highlighting region-specific regulatory modules involving lncRNAs and signaling-related genes [42, 44, 55–59]**.**

These region-specific findings may be tentatively organized within a literature-supported interpretive scaffold involving metabolism, neuronal excitability, Ca^2+^ influx, and cytoskeletal remodeling [42, 54–56, 58–60]. This interpretation is also broadly consistent with neuropathological evidence linking tau-related processes more closely than amyloid burden to regional atrophy and cognitive decline in ad [61–63]. However, these biological relationships should be viewed as literature-informed interpretations of the detected gene–brain region associations, rather than as direct mechanistic outputs of the present statistical framework. Figure 10 summarizes this proposed interpretation in a region-dependent manner. Methodologically, NL-HSIM(O) showed superior or comparable performance relative to SKAT and aSPU across the simulation settings considered, with particular advantages under nonlinear multi-signal architectures. Thus, its main strength lies in providing inferentially valid and flexible modeling when the underlying association structure may deviate from simple additive linear effects. From a translational perspective, the identified region-linked modules may serve as candidates for downstream functional validation. The proposed framework is designed for individual-level genotype and phenotype data and is not intended to replace summary-statistics-based GWAS methods, which remain indispensable for large-scale meta-analysis and privacy-constrained settings. Instead, NL-HSIM(O) should be viewed as a complementary secondary-analysis tool for deeply phenotyped cohorts in which subject-level data are available. This design is important because several steps of the framework—including DC-SIS screening, kernel construction, KPCA, and de-sparsified LASSO inference—are intrinsically defined at the subject level and cannot be reconstructed from conventional marginal SNP-level summary statistics alone. Such an individual-level formulation may be particularly useful when the genetic architecture departs from simple additive marginal effects, e.g. through nonlinear threshold effects, dominance, epistasis, or subject-level similarity structures.

Several limitations should also be noted. First, the current implementation relies on Gaussian kernels, motivating future work on adaptive or multi-kernel extensions. Second, covariates are not jointly kernelized with SNPs; this separation is intentional because joint nonlinear kernelization could amplify covariate main effects and induce spurious covariate–genotype interactions, potentially biasing downstream inference. Third, although the Nyström approximation substantially reduces computational burden, KPCA stability in smaller samples and algorithmic scalability in larger cohorts still require further empirical study. Within the chromosome-wide simulation settings considered, this additional computational cost was accompanied by improved power, particularly under nonlinear multi-signal architectures (Fig. 5; Supplementary Fig. S8). Finally, gene-level FDR control may obscure modest polygenic signals, motivating future work on more sensitive *P*-value aggregation as well as integrative extensions involving multi-omics data, longitudinal imaging, and functional validation.

**Key Points**

- The proposed NL-HSIM(O) method integrates Kernel Principal Component Analysis (KPCA) with de-sparsified LASSO to address nonlinear gene effects and dependencies in high-dimensional genomic data, overcoming limitations of traditional linear GWAS approaches.
- We implemented the Nyström approximation to accelerate KPCA computations, reducing memory requirements from O(*n*^2^) to O(*nm*) (with *m* ≪ *n*), significantly improving computational efficiency.
- We employed two principal component retention strategies and combined the advantages of NL-HSIM(A) and NL-HSIM(PA) through omnibus testing, ultimately obtaining NL-HSIM(O).
- A threshold effect model was utilized to construct nonlinear effects for SNP data. The statistical power of NL-HSIM(O) was demonstrated to be superior to Linear-HSIM.
- The proposed methodology can be extended to other group-wise association tests, including pathway association analyses.

## Supplementary Material

supplementary_file_bbag275

## Data Availability

The clinical data used in this study were obtained from the ADNI database (https://adni.loni.usc.edu/). Access to ADNI data is available to qualified researchers upon application and approval by the ADNI Data Sharing and Publications Committee. The simulation data analyzed in this study can be generated using the procedures and code provided by the authors.

## Software availability

The software implementation of the proposed NL-HSIM(O) method, including all scripts required to reproduce the analyses, is publicly available at https://github.com/zhangzechen123/NL-HSIM.

## Notes

Haitao Yang is a professor in the School of Public Health at Hebei Medical University. His research interest is to develop and apply novel statistical methods to solve genetics and genomics questions related to complex diseases.
