# Supplementary material for: Nonlinear kernel-based high-dimensional inference for set-based genetic association studies
Source: Brief Bioinform. 2026 May 27;27(3):bbag275. doi: 10.1093/bib/bbag275 (PMC13215594; doi:10.1093/bib/bbag275)
Supplement: supplementary_file_bbag275 [file supplementary_file_bbag275.docx]

Supplemental file for “**Nonlinear kernel-based high-dimensional inference for set-based genetic association studies**”

Zechen Zhang[
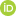
](https://orcid.org/0000-0002-5526-5255)¹,²,³, Hui Yang¹,²,³, Meilin Zhu¹, Ran Guo¹, Fuzhao Chen¹, Hui Dong^4^, Yuehua Cui[
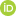
](https://orcid.org/0000-0003-4374-0909)^5^,*, Haitao Yang[
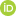
](https://orcid.org/0000-0003-4374-0909)¹,²,³,*

¹Division of Health Statistics, School of Public Health, Hebei Medical University, 361 East Zhongshan Road, Shijiazhuang, Hebei 050017, P.R. China

²Hebei Key Laboratory of Environment and Human Health, 361 East Zhongshan Road, Shijiazhuang, Hebei 050017, P.R. China

³Hebei Key Laboratory of Forensic Medicine, 361 East Zhongshan Road, Shijiazhuang, Hebei 050017, P.R. China

^4^Department of Neurology, Second Hospital of Hebei Medical University, 215 West Heping Road, Shijiazhuang, Hebei 050000, P.R. China

^5^Department of Statistics and Probability, Michigan State University, 619 Red Cedar Road, East Lansing, MI 48824, USA.

*Corresponding authors. Haitao Yang, Division of Health Statistics, School of Public Health, Hebei Medical University, 361 East Zhongshan Road, Shijiazhuang, Hebei 050017, P.R. China. Tel (Fax): 86-0311-86266991; E-mail: [haitaoyang@hebmu.edu.cn](mailto:haitaoyang@hebmu.edu.cn); Yuehua Cui, Department of Statistics and Probability, Michigan State University, East Lansing, MI 48824, United States. Tel: (517)432-7098, Fax: (517)432-1405; E-mail: cuiy@msu.edu

Key words: kernel method, nonlinear high-dimensional inference, *p*-value combination, omnibus test, SNP–set association

This supplemental file includes some methodological details (Section I) as well as additional simulation analysis results (Section II). Additional proofs and implementation details are provided where appropriate.

**Section I**

**Distance correlation and its notation**

Following standard expositions on distance correlation, we use it as a metric of statistical dependence between random vectors $\mathbf{X}\in\mathbb{R}^{p}$ and$\mathbf{Y}\in\mathbb{R}^{q}$, which is sensitive to both linear and nonlinear associations[1]. The squared distance covariance is defined via characteristic functions as:

${d\mathrm{cov}}^{2}\left( \mathbf{X,Y} \right)={\int_{\mathbb{R}^{dx+dy}}\left| \phi_{\mathbf{X,Y}}\left( \mathbf{t,s} \right)-\phi_{\mathbf{X}}\left( \mathbf{t} \right)\phi_{\mathbf{Y}}\left( \mathbf{s} \right) \right|}^{2}w(\mathbf{t,s})d\mathbf{t}d\mathbf{s}$ (S1)

Let $\phi_{\mathbf{X}}(\mathbf{t})$, $\phi_{\mathbf{Y}}(\mathbf{s})$ be the respective characteristic functions of the random vectors **X** and **Y**, and $\phi_{\mathbf{X,Y}}(\mathbf{t,s})$ is the joint characteristic function of **X** and **Y**.

The weight function is defined as $w(\mathbf{t,s})={\{c_{dx}c_{dy}\|\mathbf{t}{\|}_{dx}^{1+dx}\|\mathbf{s}{\|}_{dy}^{1+dy}\}}^{-1}$with$c_{d}=2\pi^{(1+d\text{)}/2}/\Gamma\{(1+d)/2\}$, ${\|\mathbf{a}\|}_{d}$stands for the Euclidean norm of $\mathbf{a}\in\mathbb{R}^{d}$.

Distance correlation (DC) between **X** and **Y** is then defined as

$\text{dcorr}\text{(}\text{X,Y}\text{)}=\frac{\mathrm{dcov}(\mathbf{X,Y})}{\sqrt{\mathrm{dcov}(\mathbf{X,X})\mathrm{dcov}(\mathbf{Y,Y})}}$ (S2)

In practice we estimate dcorr using the unbiased U-statistic, built from double-centered pairwise distance matrices of **X** and **Y**. This quantity is used to rank SNPs in DC-SIS prior to the downstream kernel analysis.

**Nyström Approximation for Kernel Methods**

In kernel methods, the computational complexity increases quadratically with the sample size ***n****,* which can be prohibitive for large datasets. To mitigate this challenge, we employ the Nyström method[2], which approximates the kernel matrix by using a subset of the data. This approximation reduces the complexity from *O*(*n*^3^) to *O*(*nm^2^*), significantly improving computational efficiency while maintaining a reasonable level of accuracy.

The Nyström approximate step is as follows, suppose **X** is an *n*×*p* matrix of original observation data, singular value decomposition (SVD) of **X** leads to

$\mathbf{X}=\mathbf{U}\boldsymbol{\Sigma}^{1/2}\mathbf{V}^{T}.$ (S3)

Using the linear kernel function as an example, we can create a kernel matrix based on **X**:

$\mathbf{K}=\mathbf{X}\mathbf{X}^{T}=\mathbf{U}\boldsymbol{\Sigma}^{1/2}\mathbf{V}^{T}\mathbf{V}\boldsymbol{\Sigma}^{1/2}\mathbf{U}^{T}$ (S4)

where **V** is an orthogonal matrix, so $\mathbf{V}^{T}\mathbf{V}$ is an identity matrix **I**, which gives

$\mathbf{K}=\boldsymbol{U\Sigma}\mathbf{U}^{T}$. (S5)

Assuming that **X***_m_* is an *m*×*p* data matrix (*m*<<*n*), the SVD of **X***_m_* yields

$\mathbf{X}_{m}=\mathbf{U}_{m}\boldsymbol{\Sigma}_{m}^{1/2}\mathbf{V}_{m}^{T}$, from which it is further obtained that:

$\mathbf{V}_{m}=\mathbf{X}_{m}^{T}\mathbf{U}_{m}\boldsymbol{\Sigma}_{m}^{-1/2}$. (S6)

Also, because $\mathbf{X}=\mathbf{U}\boldsymbol{\Sigma}^{1/2}\mathbf{V}^{T}$, from this, we can further obtain: $\mathbf{U}=\mathbf{XV}\boldsymbol{\Sigma}^{-1/2}$. By replacing **V** with $\mathbf{V}_{m}=\mathbf{X}_{m}^{T}\mathbf{U}_{m}\boldsymbol{\Sigma}_{m}^{-1/2}$and $\boldsymbol{\Sigma}$ with $\boldsymbol{\Sigma}_{m}$, respectively, an approximation of **U** can be obtained $\tilde{\mathbf{U}}$,

$\tilde{\mathbf{U}}=\mathbf{X}\mathbf{X}_{m}^{T}\mathbf{U}_{m}\boldsymbol{\Sigma}_{m}^{-1/2}\boldsymbol{\Sigma}_{m}^{-1/2}=\mathbf{X}\mathbf{X}_{m}^{T}\mathbf{U}_{m}\boldsymbol{\Sigma}_{m}^{-1}$. (S7)

Using $\tilde{\mathbf{U}}$ and $\boldsymbol{\Sigma}_{m}$, we can obtain ${\tilde{\mathbf{K}}}_{n\times n}$,

$\begin{aligned} {\tilde{\mathbf{K}}}_{n\times n}=\tilde{\mathbf{U}}\boldsymbol{\Sigma}_{m}\mathbf{U}^{T} \\ \text{ }\text{ }=\mathbf{X}\mathbf{X}_{m}^{T}\mathbf{U}_{m}\boldsymbol{\Sigma}_{m}^{-1}\boldsymbol{\Sigma}_{m}{(\mathbf{X}\mathbf{X}_{m}^{T}\mathbf{U}_{m}\boldsymbol{\Sigma}_{m}^{-1})}^{T} \\ \text{ }\text{ }=\mathbf{X}\mathbf{X}_{m}^{T}\mathbf{U}_{m}{(\mathbf{X}\mathbf{X}_{m}^{T}\mathbf{U}_{m}\boldsymbol{\Sigma}_{m}^{-1})}^{T} \\ \text{ }\text{ }=\mathbf{X}\mathbf{X}_{m}^{T}\{\mathbf{U}_{m}\left( \boldsymbol{\Sigma}_{m}^{-1} \right)^{T}\mathbf{U}_{m}^{T}\}{(\mathbf{X}\mathbf{X}_{m}^{T})}^{T} \\ \text{ }\text{ }=\mathbf{K}_{n\times m}\mathbf{K}_{m\times m}^{\dagger}\mathbf{K}_{n\times m}^{T} \end{aligned}$ (S8)

If and only if $\mathbf{K}_{m\times m}$ is a symmetric positive definite matrix, its SVD decomposition is consistent with the eigenvalue decomposition, that is,

$\mathbf{K}_{m\times m}=\mathbf{U}_{m}\boldsymbol{\Sigma}_{m}\mathbf{U}_{m}^{T}=\mathbf{P}_{m}\mathbf{Q}_{m}\mathbf{P}_{m}^{-1}$,

then ${\tilde{\mathbf{K}}}_{n\times n}$ can also be written as follows:

$\begin{aligned} {\tilde{\mathbf{K}}}_{n\times n}=\mathbf{K}_{n\times m}\mathbf{P}_{m}\mathbf{Q}_{m}\mathbf{P}_{m}^{-1}\mathbf{K}_{n\times m}^{T} \\ \text{ }\text{ }=\mathbf{K}_{n\times m}\mathbf{P}_{m}\mathbf{Q}_{m}^{-1/2}\mathbf{Q}_{m}^{-1/2}\mathbf{P}_{m}^{-1}\mathbf{K}_{n\times m}^{T} \end{aligned}$ (S9)

Let $\mathbf{G}=\mathbf{K}_{n\times m}\mathbf{P}_{m}\mathbf{Q}_{m}^{-1/2}$, then $\mathbf{Q}_{m}^{-1/2}\mathbf{P}_{m}^{-1}\mathbf{K}_{n\times m}^{T}=\mathbf{G}^{T}$, so that ${\tilde{\mathbf{K}}}_{n\times n}=\mathbf{G}\mathbf{G}^{T}$.

**A brief introduction of the Pre-image method**

The **Pre-image method** is a critical technique in Kernel Principal Component Analysis (KPCA), particularly when working with nonlinear kernels, such as the Gaussian kernel. The goal of the pre-image method is to find the corresponding point in the original data space that maps to a given point in the feature space, which is inherently nonlinear and often not directly invertible.

Mika et al. proposed an iterative method[3] for estimating the pre-image of a feature vector *z* in the kernel-induced feature space. The problem is formulated as a nonlinear optimization problem:

$x*=\underset{z\in\chi}{\mathrm{argmin}}{\|\Phi(z)-\varphi\|}_{H}^{2}=\underset{z\in\chi}{\mathrm{argmin}}(\langle\Phi(z),\Phi(z)\rangle-2\langle\Phi(z),\varphi\rangle+\langle\varphi,\varphi\rangle)$ (S10)

As $\varphi=\Sigma_{i=1}^{N}\gamma_{i}\Phi(x_{i})$, we have

$\langle\varphi,\varphi\rangle=\langle\overset{N}{\underset{i=1}{\Sigma}}\gamma_{i}\Phi(x_{i}),\overset{N}{\underset{i=1}{\Sigma}}\gamma_{i}\Phi(x_{i})\rangle=\gamma^{T}K\gamma$ (S11)

Therefore, $\langle\varphi,\varphi\rangle$ is independent of *Z*. After normalizing the kernel *k* such that $\langle\Phi(z),\Phi(z)\rangle=k(z,z)$is constant for all *z*, instead of minimizing Eq. (S10), we can reformulate the problem as a maximization problem:

$x*=\underset{z\in\chi}{\mathrm{argmax}}\langle\Phi(z),\varphi\rangle$ (S12)

From the representer theorem[4], any feature vector obtained by a kernel method can be expressed in a specific form: $\varphi=\Sigma_{i=1}^{N}\gamma_{i}\Phi(x_{i})$, substituting this expression into Eq.(S12), we obtain the following optimization problem:

$\begin{aligned} x^{*}=\underset{z\in\chi}{\mathrm{argmax}}\left\langle\Phi\left( z \right),\Sigma_{i=1}^{N}\gamma_{i}\Phi\left( x_{i} \right) \right\rangle\\ =\underset{z\in\chi}{\mathrm{argmax}}\Sigma_{i=1}^{N}\gamma_{i}\langle\Phi(z),\Phi(x_{i})\rangle\\ =\underset{z\in\chi}{\mathrm{argmax}}\Sigma_{i=1}^{N}\gamma_{i}k\left( z,x_{i} \right) \\ =\underset{z\in\chi}{\mathrm{argmax}}F(z) \end{aligned}$ (S13)

The optimization problem can be addressed using gradient descent techniques. Specifically, for a Gaussian kernel, we can employ the fixed-point iteration method. By taking the derivative of $F\left( z \right)$ with respect to$z$and setting it to zero, we arrive the following equation

$\begin{aligned} \frac{\partial F}{\partial z}=\frac{\partial}{\partial z}\sum_{i=1}^{N} \gamma_{i}k(z,x_{i})=0, \\ \Rightarrow z=\frac{\sum_{i=1}^{N} \gamma_{i}exp(-\left\| z-x_{i} \right\|^{2}/(2\sigma^{2}))x_{i}}{\sum_{i=1}^{N} \gamma_{i}exp(-\left\| z-x_{i} \right\|^{2}/(2\sigma^{2}))} \end{aligned}$ (S14)

By the fixed-point iteration theorem,$x*$is a convergent point of the sequences $\{z_{n}\}$

$\Rightarrow z_{n+1}=\frac{\sum_{i=1}^{N} \gamma_{i}exp(-\left\| z-x_{i} \right\|^{2}/(2\sigma^{2}))x_{i}}{\sum_{i=1}^{N} \gamma_{i}exp(-\left\| z-x_{i} \right\|^{2}/(2\sigma^{2}))}$ (S15)

**Pre-image Reconstruction via Fixed-point Iteration**

To ensure generalizability, we perform ten-fold cross-validation by randomly partitioning the dataset into training (90%) and test (10%) subsets. KPCA is applied to the training set to derive kernel principal components (KPCs), and reconstruction performance is evaluated on the test set. The training data are first mapped to the kernel-induced feature space, where PCA is performed for dimensionality reduction.

The RE is computed as the mean Euclidean distance between reconstructed and original test data:

$RE=\frac{1}{n_{test}}\sum_{i=1}^{n_{test}} \left\| X_{test,i}-Z_{test,i} \right\|_{F}^{2}$ (S16)

where *X_test,i_* is the original test data sample, and Z*_test,i_* is the corresponding reconstructed sample obtained by mapping the kernel PCA representation back to the input space.

A grid search is performed over candidate KPC counts (ranging from 1 to 0.9×nₜᵣₐᵢₙ) to identify the optimal number minimizing the reconstruction error (RE) via ten-fold cross-validation (Table S1). The number of KPCs corresponding to the lowest average RE is selected as optimal.

For pre-image reconstruction in Gaussian KPCA, the choice of the initial guess $z_{0}$of the pre-image in the input space plays a critical role in determining whether Mika’s iterative algorithm converges. As a simple yet effective strategy, we initialize this estimate using the column-wise mean of the test samples in each fold:

$z_{0}=colMeans(\mathbf{X}_{i})$, where $colMeans(\cdot)$ is the function that calculates the column means of a matrix. $\mathbf{X}_{i}$denotes the input-space test set in the *i*-th fold[4]. This initialization provides a neutral starting point near the data manifold, facilitating stable convergence.

**A brief introduction of the de-sparsified LASSO**

In high-dimensional settings (𝑝≫𝑛), the LASSO provides stable estimation and variable screening, but its coefficient estimates are biased due to penalization and the lack of exact orthogonality between the target regressor and the remaining covariates. To enable valid inference (e.g., component-wise *p*-values) in this regime, we adopt the de-sparsified (debiased) LASSO framework, which corrects the LASSO bias and yields asymptotically normal test statistics[5-7].

Table S1 Pseudocode: KPCA with Pre-image Error Minimization

| **Input:**   - Dataset $D=\{\mathbf{X}_{1},\mathbf{X}_{2},\ldots,\mathbf{X}_{n}\}\in\mathbb{R}^{m}$—*A dataset consisting of n samples, each with m features*. - Set of kernels $\{k_{p_{1}},k_{p_{2}},\ldots,k_{p_{0.9n}}\}$ *represents a KPCA configuration that retains h principal components p*. - Threshold $\delta=1\times{10}^{-5}$ —*A convergence threshold for the iterative pre-image reconstruction procedure*.   **Initialize:**  Set $h\text{ := 1}$ **Procedure:**  1. **Repeat until** *h*>0.9*n*:  1.1 Set *i*:=1  1.2 **For each** *i*=1 to 10: (*i* indexes the *i* -th fold in 10-fold cross-validation):  a. Exclude **X***_i_* from *D*, define $D^{(-i)}=D\setminus\{\mathbf{X}_{i}\}$ b. Perform KPCA on $D^{(-i)}$ with kernel $k_{h}$ c. Compute the pre-image $Z_{i}^{(h)}$of **X***_i_* via iterative method  - Stop iteration when $\Vert Z_{t+1}-Z_{t}\Vert<\delta$ d. Compute reconstruction error:$E_{i}^{(h)}={\Vert\mathbf{X}_{i}-\mathbf{Z}_{i}^{(h)}\Vert}^{2}$  1.3 Compute 10-fold cross-validation error:$E^{(h)}=\frac{1}{10}\sum_{i=1}^{10} E_{i}^{(h)}$  1.4 Update *h***:=***h***+**1  **Output:**  $h_{\text{opt}}:=\arg\min_{h}E^{(h)}$—*The number of retained components that minimizes average reconstruction error.*  $P_{\text{opt}}:=p_{h_{\text{opt}}}$—*Final projection/embedding output from KPCA under h_opt_*. |
| --- |

In our framework, the original high-dimensional predictors are first mapped into kernel principal components (KPCs). Let the aggregated KPC design matrix be denoted by **K**,and we model the relationship between the phenotype **Y** and KPCs via the linear regression in Eq.(S17).

$\mathbf{Y}=\mathbf{K}\beta+\epsilon$ (S17)

The LASSO is then fitted on **Y** and **K** to obtain an initial estimate of the coefficient vector.

To perform inference on a specific KPC coefficient, we need a direction that approximately removes the effect of the remaining KPCs. For the *j*-th KPC, we first compute the LASSO residual **Z***_j_* by regressing **K***_j_* (the j-th column of **K**) on all other KPCs **K***_-j_*, $\mathbf{Z}_{j}=\mathbf{K}_{j}-\mathbf{K}_{-j}\hat{\gamma}_{\text{Lasso}}^{(j)}$,where $\hat{\gamma}_{\text{Lasso}}^{(j)}$ is the LASSO estimator obtained by solving:

$\hat{\gamma}_{\text{Lasso}}^{(j)}=\arg\min_{\gamma}\{n^{-1}{\|\mathbf{K}_{j}-\mathbf{K}_{-j}\gamma\|}_{2}^{2}+\lambda_{j}{\|\gamma\|}_{1}\}$ (S18)

The de-sparsified LASSO estimator for $\hat{\beta}_{j}$ is then constructed as:

$\hat{\beta}_{j}=\frac{Z_{j}^{T}Y}{Z_{j}^{T}K_{j}}-\Sigma_{k\neq j}\frac{Z_{j}^{T}X_{k}}{Z_{j}^{T}K_{j}}\hat{\beta}_{LASSO,k}$ (S19)

Where $\hat{\beta}_{LASSO,k}$ is the LASSO estimate of the *k*-th coefficient from the regression of **Y** on the full set of predictors **K**. This estimator corrects the bias inherent in standard LASSO through the second term. Under mild regularity conditions[5] for high-dimensional inference, $\hat{\beta}_{j}$ follows an asymptotic normal distribution:

$\frac{\sqrt{n}(\hat{\beta}_{j}-\beta_{j}^{0})}{\sigma_{\varepsilon}\sqrt{\Omega_{jj}}}\to N(0,1)\text{ as }d\geq n\to\infty$ (S20)

where $\Omega_{jj}=\frac{n\mathbf{Z}_{j}^{T}\mathbf{Z}_{j}}{(\mathbf{K}_{j}^{T}\mathbf{Z}_{j})(\mathbf{K}_{j}^{T}\mathbf{Z}_{j})}$ and the *p*-value for testing $H_{0}:\beta_{j}=0$ is computed as:

$p_{j}=2(1-\Phi(|\sqrt{n}\hat{\beta}_{j}/\hat{\sigma}\sqrt{\hat{\Omega}_{jj}}|))$ (S21)

In our downstream analysis, these component-wise *p*-values are further used to derive gene-level evidence in the proposed framework.

**Section Ⅱ**

**Simulation studies**

**Case Ⅰ: simulation with discrete variants**

Data were generated from an effect function regression model, i.e.,

$$Y=h(X)\beta+\varepsilon$$

where the *h*(*X*) is the effect function of predictors, *β* is the effect coefficient vector, and$\varepsilon\sim N(0,1)$, *X*=(*X*_G1_, *X*_G2_) represent predictors in two groups (G1 with 100 predictors and G2 with 700 predictors). Each group of predictors was generated from a multivariate normal distribution with mean 0 and covariance $\Sigma=(\sigma_{ij})$, where $\sigma_{ij}=\rho^{|i-j|}$. This AR(1) structure was adopted as a simplified synthetic dependence model to provide a tractable baseline for controlled method evaluation, and was not intended to fully reproduce the block-like linkage disequilibrium architecture observed in real human genetic data. The generated continuous values were then discretized into three ordered categories (0, 1, 2) using the empirical 33% and 67% quantiles to create a controlled discrete-predictor setting for method evaluation. This discretization was not intended to reproduce genotype frequencies under Hardy–Weinberg equilibrium. In each group, there were both signals and noises. Let

*β*_G_=(*c*,…,*c*,0,…,0)

denote the effect coefficient vector in group *G*, where *c* represents the non-zero coefficient. Across different scenarios, the specification of $h(\cdot)$ and *β*_G_ are as follows:

In nonlinear scenarios, we set $h(X)=\cos(\pi X^{2})$; in linear scenarios, we used the identity mapping *h*(*X*) = *X*. The powers for G1 and G2 were calculated based on *β*_G_ (G=1, 2).

In CWSM setting, we specified

$$\beta_{1}=(\underset{15}{\underset{︸}{c,\ldots,c}},\underset{85}{\underset{︸}{0,\ldots,0}}),\text{ }\beta_{2}=(\underset{50}{\underset{︸}{c,\ldots,c}},\underset{650}{\underset{︸}{0,\ldots,0}})$$

For the nonlinear scenario, the signal magnitudes were set to

*c*_1_0.1,0.2,0.3}, *c*_2_0.2,0.4,0.6},

whereas for the linear scenario, we used

*c*_1_=*c*_2_0.05,0.075,0.1}.

In DSSM setting, we specified

$$\beta_{1}=(c,\underset{99}{\underset{︸}{0,\ldots,0}}),\beta_{2}=(c,c,c,\underset{697}{\underset{︸}{0,\ldots,0}})$$

for the nonlinear scenario, with

*c*_1_0.5,1,1.5}, *c*_2_1,3,5}.

For linear scenario, we specified

$$\beta_{1}=(c,\underset{99}{\underset{︸}{0,\ldots,0}}),\text{ }\beta_{2}=(c,\underset{699}{\underset{︸}{0,\ldots,0}})$$

with

*c*_1_0.1,0.3,0.5}, *c*_2_0.05,0.15,0.25}.

In all scenarios, we varied the sample size (*n*=400, 600) and the within-group correlation (*ρ*=0.2, 0.5, 0.8). In addition, the number of variables retained after screening via distance correlation was set as *d*=*n*/log(*n*). The Type I error was assessed under the null hypothesis *c*=0.

**Case II Simulation with SNP Data from Chromosome 8**

In this simulation, we evaluated the performance of group-level statistical inference for discrete predictors (SNP genotypes) under a chromosome-wide setting. To incorporate realistic linkage disequilibrium (LD) patterns while maintaining computational tractability, we used genotype data from chromosome 8 of the Alzheimer’s Disease Neuroimaging Initiative (ADNI) study. Genotypes were coded as 0, 1, or 2 (minor-allele dosage) based on the minor allele frequency (MAF).

We selected chromosome 8 because the number of SNPs contained in two genes on this chromosome, SGCZ (254 SNPs) and CSMD1 (895 SNPs), are comparable to the numbers of simulated SNPs assigned to the two groups in Case I, thereby improving consistency between the real-data-based and fully simulated settings. In total, 723 genes comprising 10,946 SNPs were extracted from chromosome 8, and SGCZ and CSMD1 were treated as focal genes for power evaluation. Following Case I, we randomly sampled 𝑛=400 and 𝑛=600 individuals without replacement to construct the simulation datasets. The phenotype was generated as,

$Y=h(X_{G})\beta_{G}+\varepsilon$, $G\in(SGCZ,CSMD1)$,

where *h*(X_G_) is the effect function defined in Case I, and *c* denotes the effect size applied to one gene at a time. Signal structures were specified under both CWSM and DSSM settings.

In CWSM, we set,

$$\beta_{SGCZ}=(\underset{15}{\underset{︸}{c_{SGCZ},\ldots,c_{SGCZ}}},\underset{239}{\underset{︸}{0,\ldots,0}}),\beta_{CSMD1}=(\underset{50}{\underset{︸}{c_{CSMD1},\ldots,c_{CSMD1}}},\underset{845}{\underset{︸}{0,\ldots,0}})$$

Where, in the nonlinear scenario, *c*_SGCZ_0.05, 0.07, 0.09} and *c*_CSMD1_0.03, 0.04, 0.05}, whereas in the linear scenario, *c*_SGCZ_0.2, 0.25, 0.3} and *c*_CSMD1_0.05, 0.075, 0.1}.

In DSSM, we set

$\beta_{SGCZ}=(\underset{1}{\underset{︸}{c_{SGCZ}}},\underset{253}{\underset{︸}{0,\ldots,0}}),\beta_{CSMD1}=(\underset{1}{\underset{︸}{c_{CSMD1}}},\underset{894}{\underset{︸}{0,\ldots,0}})$,

Where, in the nonlinear scenario, *c*_SGCZ_0.2, 0.3, 0.4} and *c*_CSMD1_0.6, 0.7, 0.8}, whereas in the linear scenario *c*_SGCZ_0.5, 0.75, 1} and *c*_CSMD1_1, 1.5, 2}.

To reduce dimensionality, we applied distance correlation (DC) screening, retaining 𝑑=𝑛/log(𝑛) variables. For each scenario, 1,000 replicates were conducted, and empirical power was calculated using FDR-corrected *p*-values after DC screening. For benchmarking, we compared the proposed NL-HSIM variants with Linear-HSIM, the Sequence Kernel Association Test (SKAT), and the adaptive sum of powered score (aSPU) test. SKAT was implemented with the linear weighted kernel and the identity-by-state (IBS) kernel, without screening, using default settings:

- kernel = "linear.weighted" or "IBS"
- method = "davies"
- weights.beta = c(1, 25)
- impute.method = "fixed"
- r.corr = 0
- max.MAF = 1

Overall, this design enables a direct comparison between the proposed framework and widely used gene-level association methods under realistic LD structures and biologically meaningful SNP distributions, while keeping the simulation setup comparable to Case I.

**Case III: simulation with quantitative predictors**

Data were generated from an effect function regression model, i.e.,

$Y=h(X)\beta+\varepsilon$,

where the $h(\cdot)$ denotes the effect function, *β* is the corresponding coefficient vector, and $\varepsilon\sim N(0,1)$. The predictor vector *X*= (*X*_G1_, *X*_G2_) consists of two groups, with G_1_ containing 100 predictors and G_2_ containing 700 predictors. Within each group, predictors follow a multivariate normal distribution with mean zero and covariance matrix$\Sigma$, where $\Sigma$ was specified as in Case I to control within-group correlation. Each group contained both signal and noise variables. Let *β*_G_=(*c*,…,*c*,0,…,0) denote the coefficient vector in group *G*, where *c* represents the non-zero effect size. For the nonlinear scenario, $h\left( \cdot\right)$ was specified as in $h(X)=X^{2}$; for the linear scenarios, we used the identity mapping *h*(*X*) =*X*. Powers for G1 and G2 were computed based on the corresponding *β*_G_ (G=1, 2).

Under the CWSM setting, we set

$$\beta_{1}=(\underset{15}{\underset{︸}{c_{1},\ldots,c_{2}}},\underset{85}{\underset{︸}{0,\ldots,0}}),\beta_{2}=(\underset{50}{\underset{︸}{c_{1},\ldots,c_{2}}},\underset{650}{\underset{︸}{0,\ldots,0}})$$

where *c*_1_0.06, 0.07, 0.08} and *c*_2_0.07, 0.08, 0.09} for the nonlinear scenario, and *c*_1_=*c*_2_0.03, 0.04, 0.05} for linear scenario.

Under the DSSM setting, we set

$\beta_{1}=(c_{1},\underset{99}{\underset{︸}{0,\ldots,0}}),\beta_{2}=(c_{2},\underset{699}{\underset{︸}{0,\ldots,0}})$,

where *c*_1_0.3, 0.4, 0.5} and *c*_2_0.4, 1.2, 2} for the nonlinear scenario, and *c*_1_=*c*_2_0.1, 0.3, 0.5} for the linear scenario.

All other settings–including sample sizes, within-group correlation levels, and the number of predictors retained after distance correlation screening–were identical to those in Case I. Type I error was assessed under the null hypothesis *c*=0.

**Performance measure**

Throughout the simulation figures, the plotted values are empirical type I error rates or power estimates computed as rejection proportions over 1000 independent replicates.

**Power comparison between NL-HSIM(O) and Linear-HSIM in the linear scenario of Case I**

Figures S1–S2 present comparisons between NL-HSIM(O) and Linear-HSIM under linear settings, along with the complete set of results for all simulation scenarios. These results indicate that although NL-HSIM(O) is specifically designed to capture nonlinear effects, it remains competitive in linear contexts as well.

**Empirical type I error control and power comparison among NL-HSIM(P), NL-HSIM(A), and NL-HSIM(PA) in Case I**

Figure S3 shows the type I error comparison in Case I with discrete predictors. NL-HSIM(P) was consistently conservative, with type I error rates well below the nominal 0.05 level across all settings. NL-HSIM(A) and NL-HSIM(PA) generally achieved rates closer to 0.05, with mild inflation observed in some configurations.


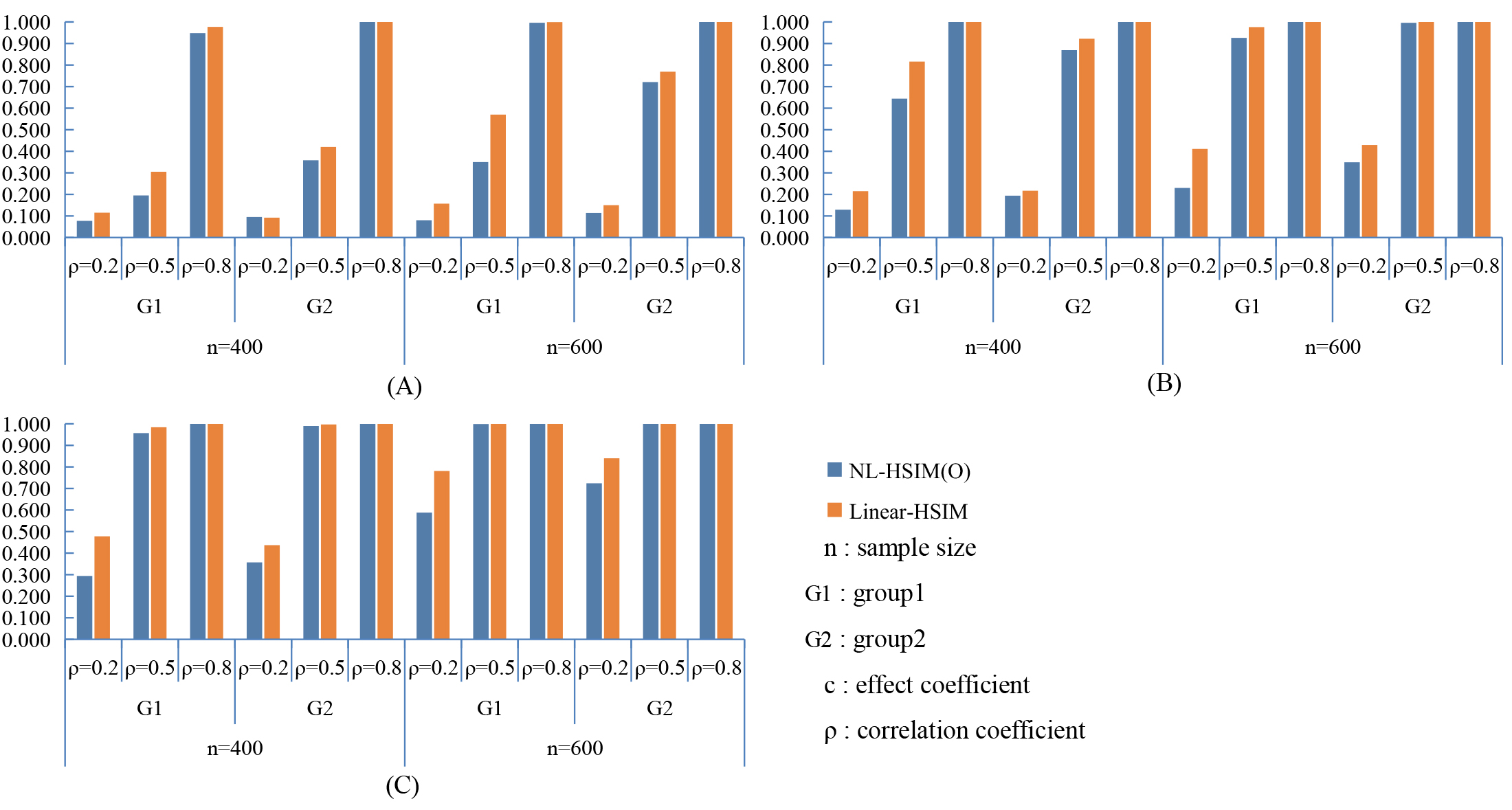


Figure S1. Comparison of NL-HSIM(O) and Linear-HSIM in linear CWSM simulation scenarios: (A) *c*_1_=*c*_2_=0.05; (B) *c*_1_=*c*_2_=0.075; (C) *c*_1_=*c*_2_=0.1.


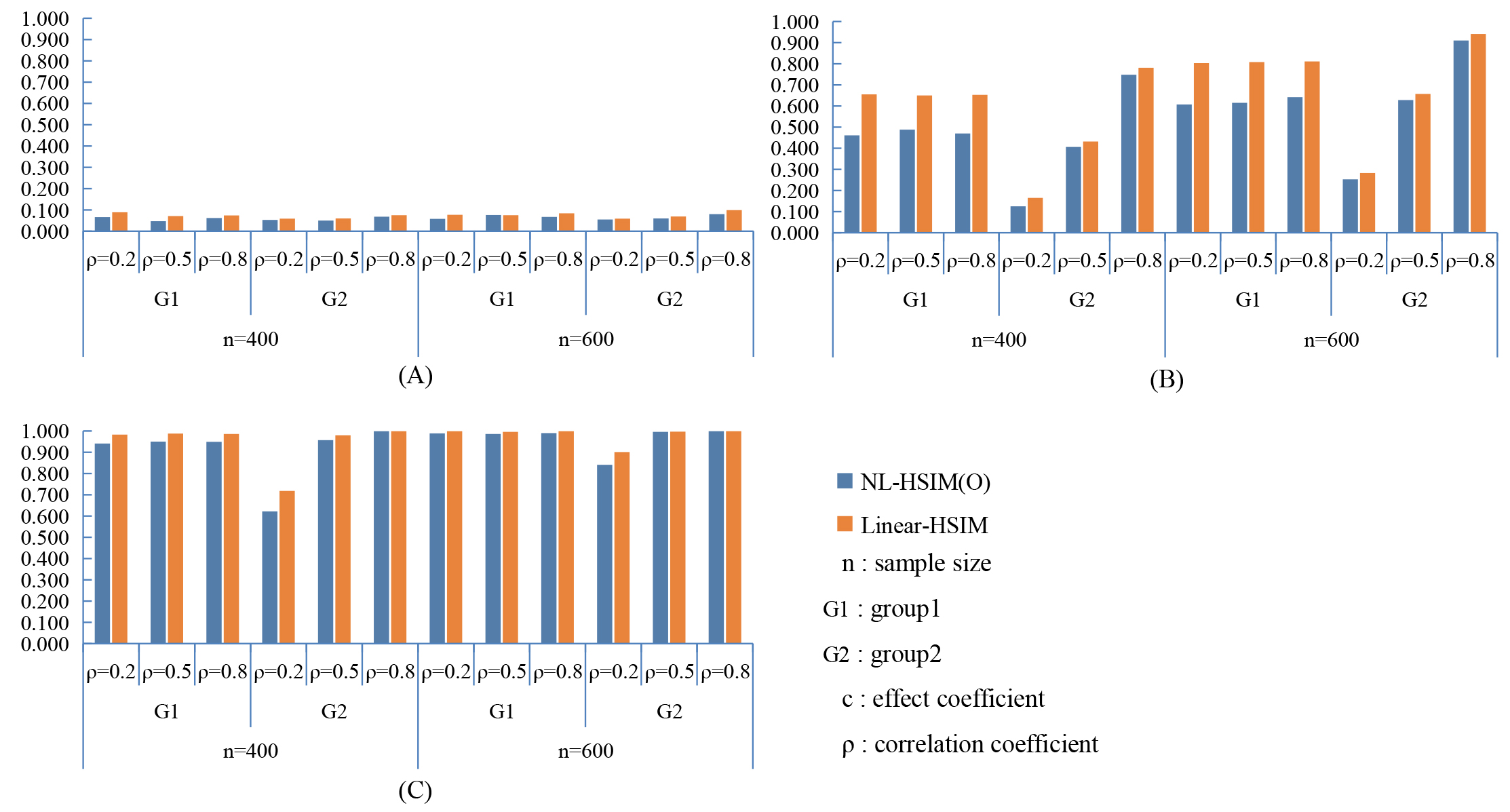


Figure S2. Comparison of NL-HSIM(O) and Linear-HSIM in linear DSSM simulation scenarios: (A) *c*_1_=0.1, *c*_2_=0.05; (B) *c*_1_=0.3, *c*_2_=0.15; (C) *c*_1_=0.5, *c*_2_=0.25.

Figures S4–S5 present the nonlinear simulation results under the CWSM and DSSM settings. In the CWSM setting (Figure S4), NL-HSIM(A) and NL-HSIM(PA) consistently outperformed NL-HSIM(P), with more pronounced differences in the larger group G2 and under weaker correlations. As signal strength increased, NL-HSIM(A) and NL-HSIM(PA) achieved near-one power, while NL-HSIM(P) remained comparatively lower. In contrast, under the DSSM setting (Figure S5), the performance gaps narrowed, and all three methods showed steadily increasing power with stronger signals, with NL-HSIM(PA) closely matching NL-HSIM(A).

In the linear scenarios (Figures S6–S7), NL-HSIM(PA) and NL-HSIM(P) showed similar power overall, with NL-HSIM(PA) having a slight advantage at higher correlations or larger sample sizes. In G1 (fewer predictors), all three methods performed similarly, whereas in G2 (more predictors), NL-HSIM(PA) maintained slightly higher power.


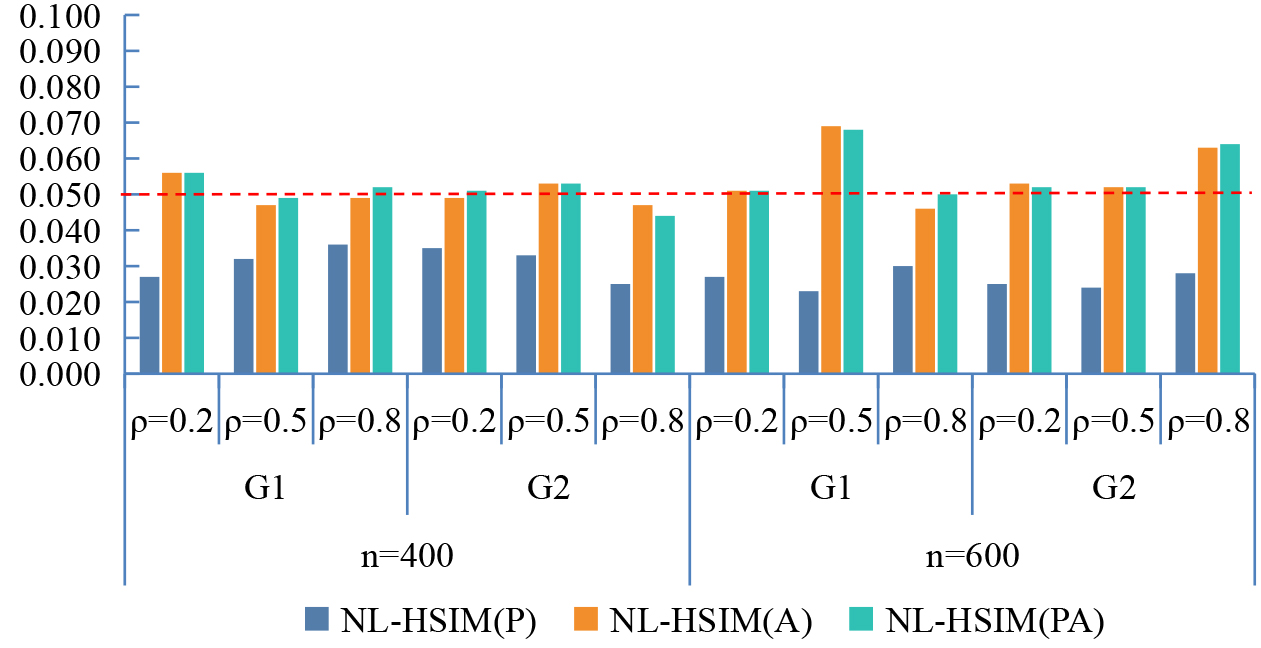


Figure S3. Comparison of empirical type I error among NL-HSIM(P), NL-HSIM(A), and NL-HSIM(PA).

Figure S4. Comparison among NL-HSIM(P), NL-HSIM(A) and NL-HSIM(PA) in Nonlinear CWSM simulation scenarios: (A)*c*_1_=0.1, *c*_2_=0.2; (B) *c*_1_=0.2, *c*_2_=0.4; (C) *c*_1_=0.3, *c*_2_=0.6.


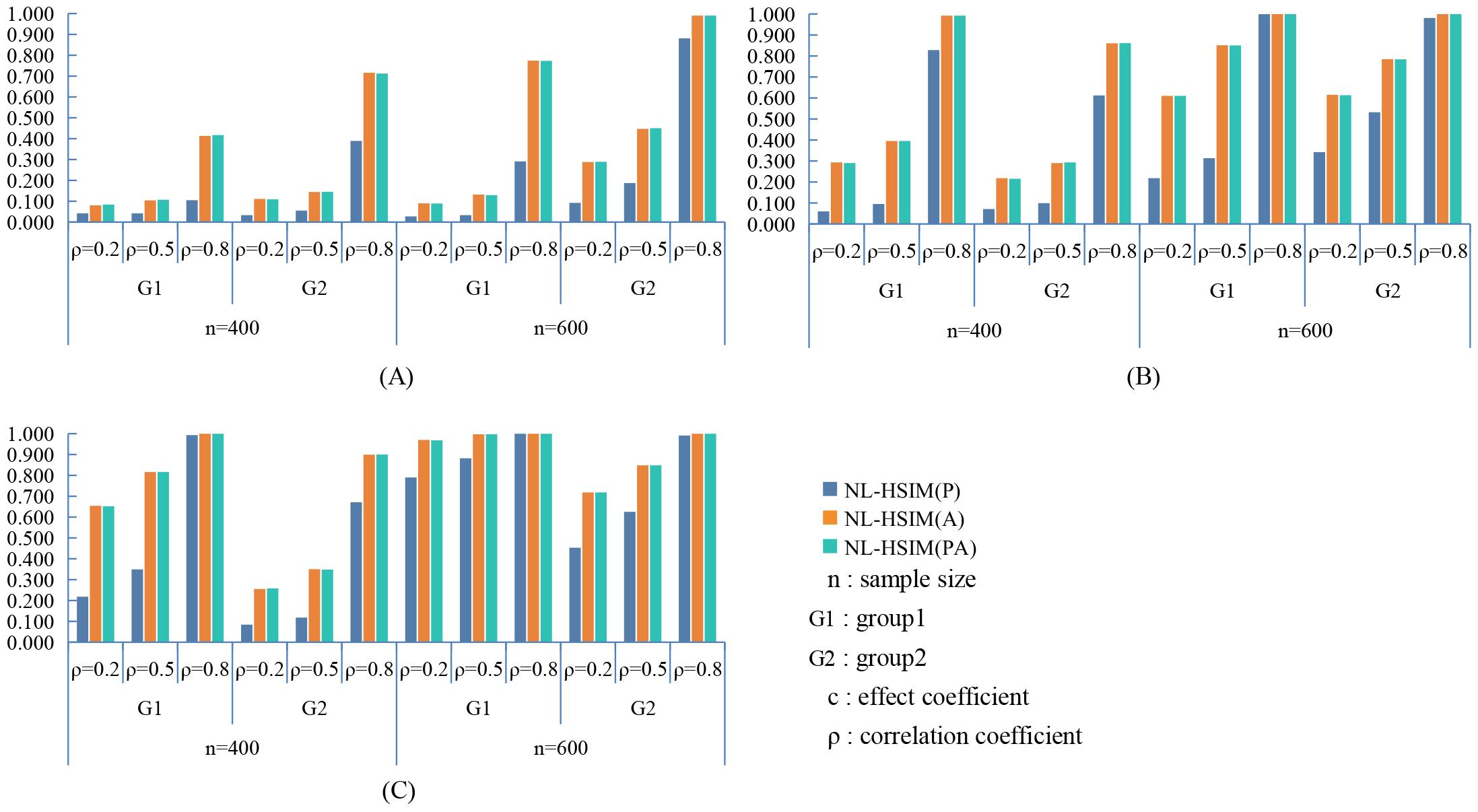

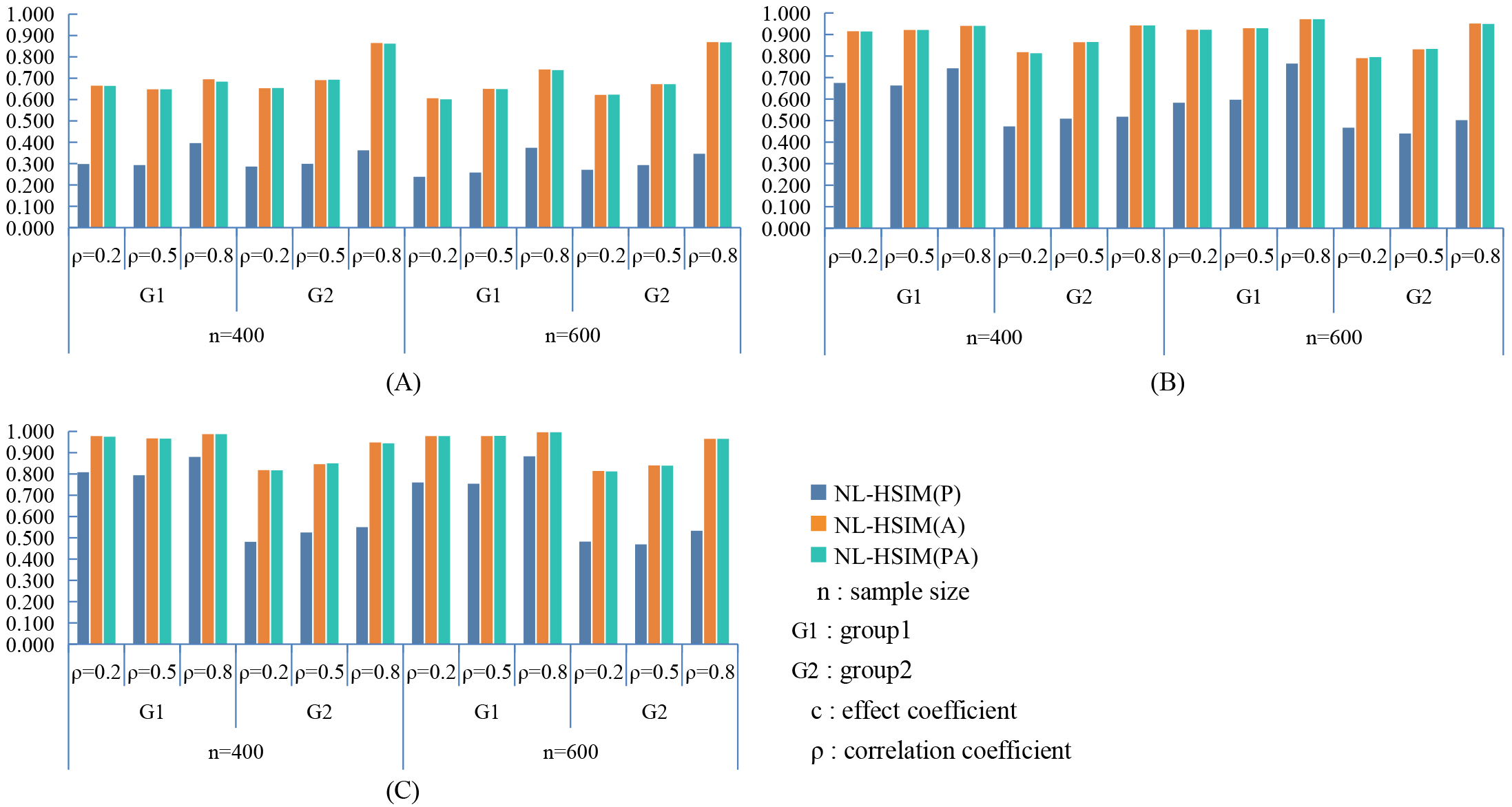


Figure S5. Comparison among NL-HSIM(P), NL-HSIM(A) and NL-HSIM(PA) in Nonlinear DSSM simulation scenarios: (A) *c*_1_=0.5, *c*_2_=1; (B) *c*_1_=1, *c*_2_=3; (C) *c*_1_=1.5, *c*_2_=5.

**Power comparison of NL-HSIM(O) with Linear-HSIM, SKAT, and aSPU in Case II**

Across the chromosome-wide SNP simulations (Figure S8), NL-HSIM(O) showed the most robust power overall and was particularly advantageous in the nonlinear settings. In the nonlinear CWSM scenario (A), NL-HSIM(O) clearly dominated for both focal genes across effect sizes, whereas SKAT(linear) remained near zero and SKAT(IBS)/aSPU provided only limited gains. In the nonlinear DSSM scenario (B), NL-HSIM(O) and aSPU achieved high power, with aSPU being especially competitive under stronger effects; SKAT(IBS) consistently outperformed SKAT(linear) but generally remained less powerful than NL-HSIM(O). In the linear scenarios (C–D), aSPU and NL-HSIM(O) attained high power as signals increased, while SKAT(IBS) showed moderate improvements and SKAT(linear) remained the weakest.

Overall, NL-HSIM(O) demonstrated a clear global advantage, achieving strong and stable power across all scenarios, correlation structures, and effect sizes, while the competing methods performed well only under specific settings (e.g., aSPU under sparse strong signals, or SKAT(IBS) under specific LD-driven configurations).

Figure S6. Comparison among NL-HSIM(P), NL-HSIM(A) and NL-HSIM(PA) in linear CWSM simulation scenarios: (A) *c*_1_=*c*_2_=0.05; (B) *c*_1_=*c*_2_=0.075; (C) *c*_1_=*c*_2_=0.1.


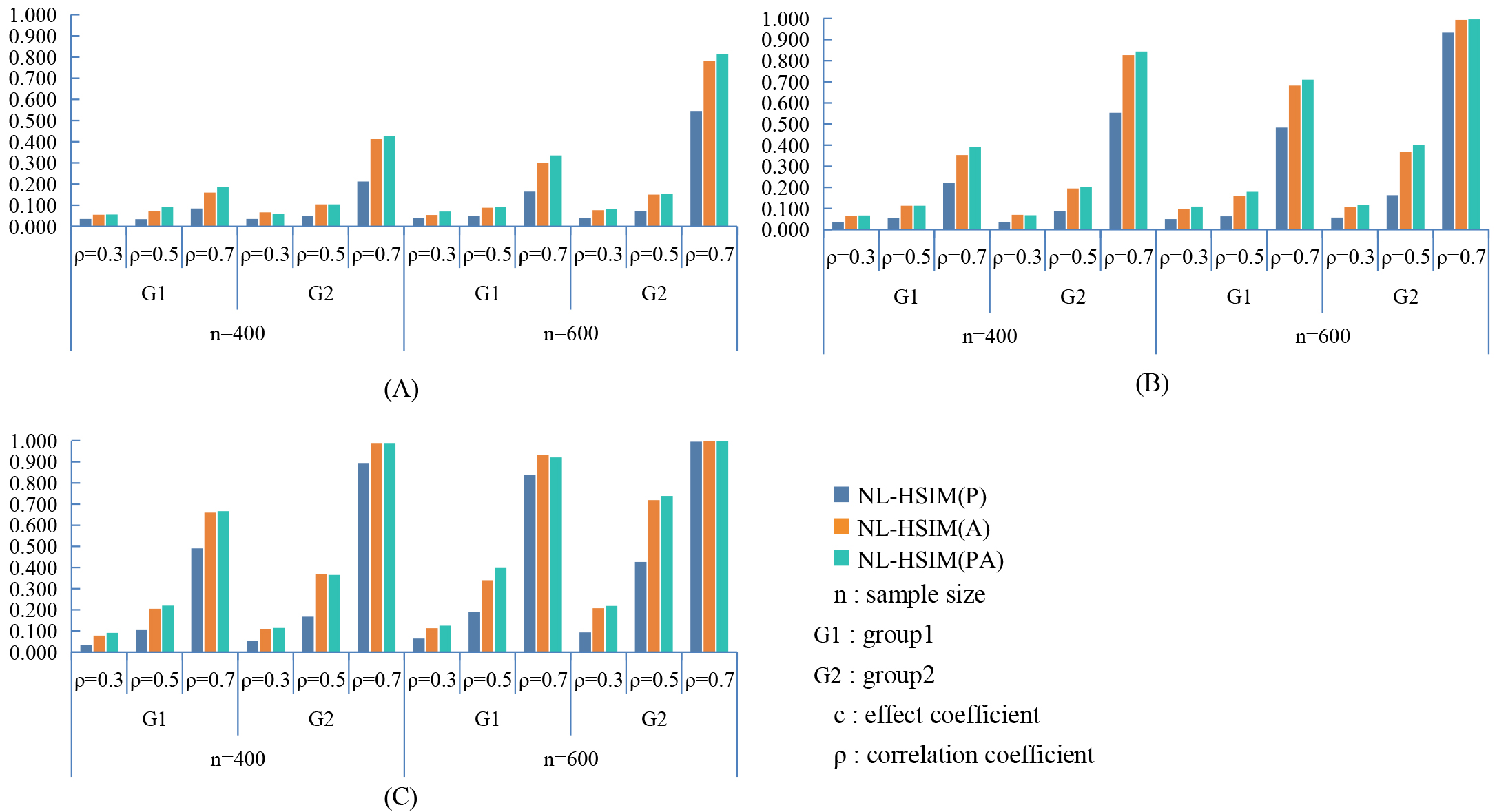


Figure S7. Comparison among NL-HSIM(P), NL-HSIM(A) and NL-HSIM(PA) in linear DSSM simulation scenarios: (A) *c*_1_=0.1, *c*_2_=0.05; (B) *c*_1_=0.3, *c*_2_=0.15; (C) *c*_1_=0.5, *c*_2_=0.25.


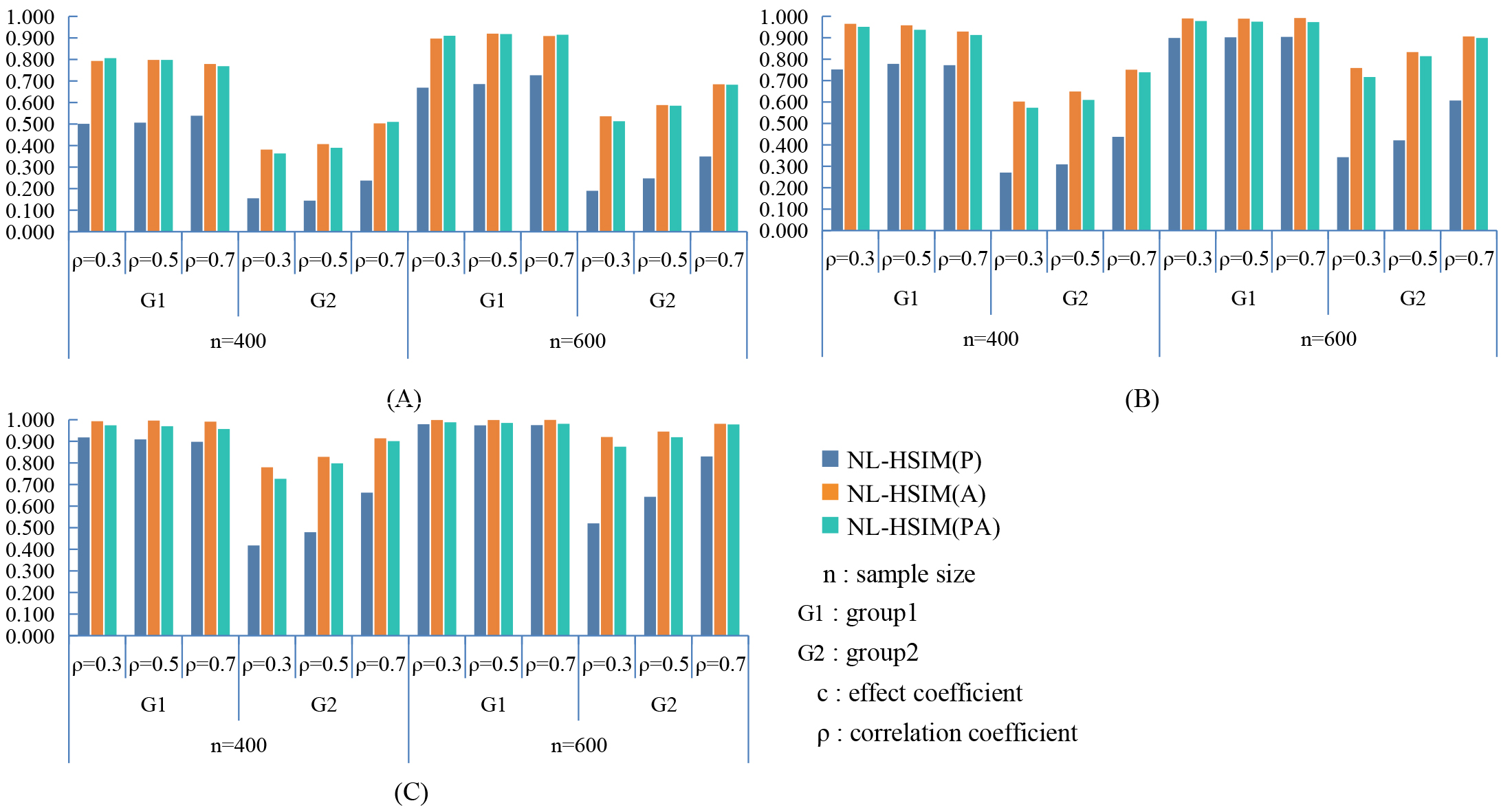

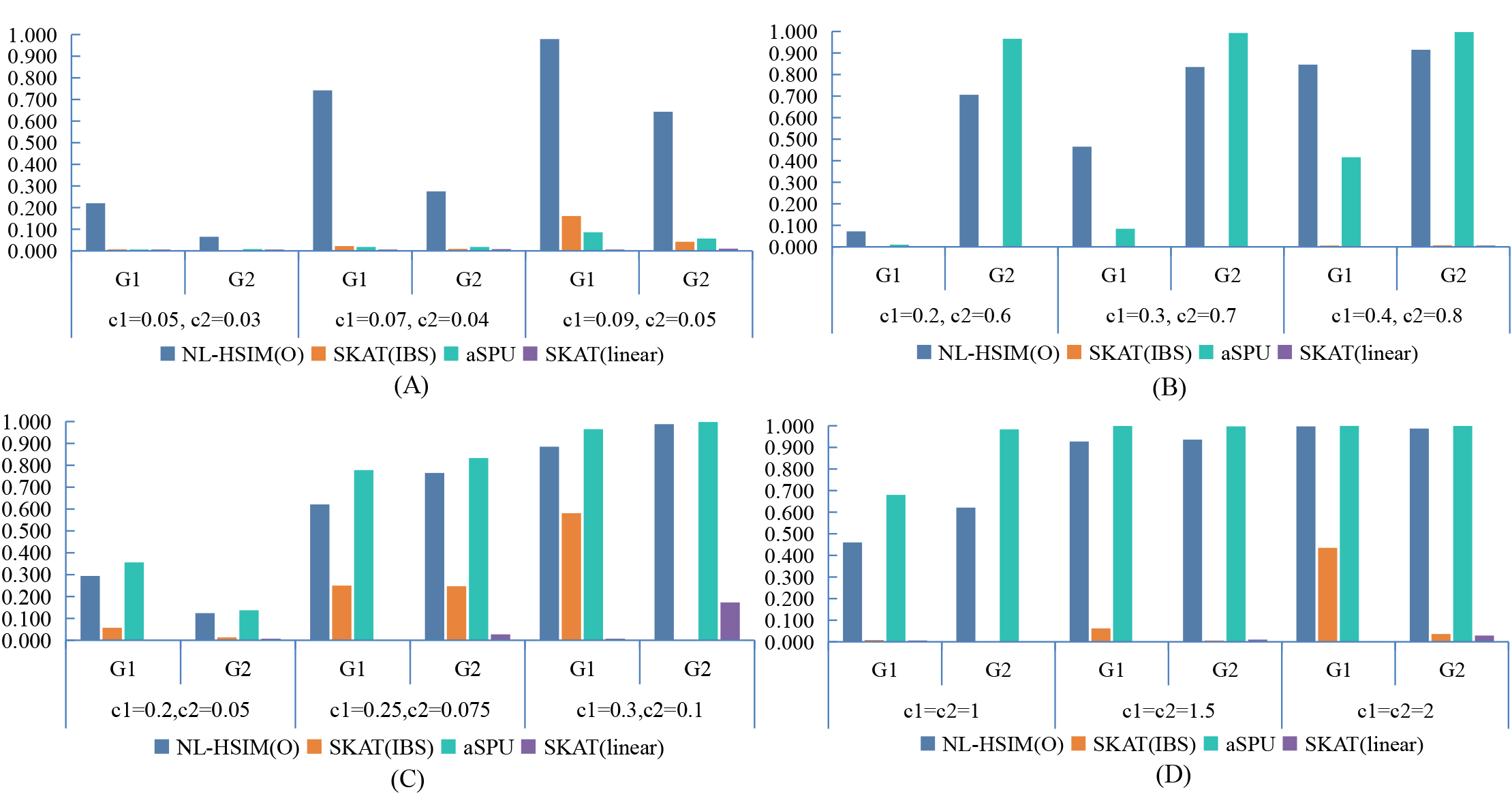


Figure S8. Power comparison of NL-HSIM(O), SKAT(IBS), aSPU, SKAT(linear) under chromosome-wide simulation scenarios with *n*=400: (A) Nonlinear CWSM; (B) Nonlinear DSSM; (C) Linear CWSM; (D) Linear DSSM.

Figure S9. Comparison of NL-HSIM(A) and NL-HSIM(PA) in different simulation settings using real SNP data from chromosome 8: (A) Nonlinear CWSM; (B) Nonlinear DSSM; (C) Linear CWSM; (D) Linear DSSM.


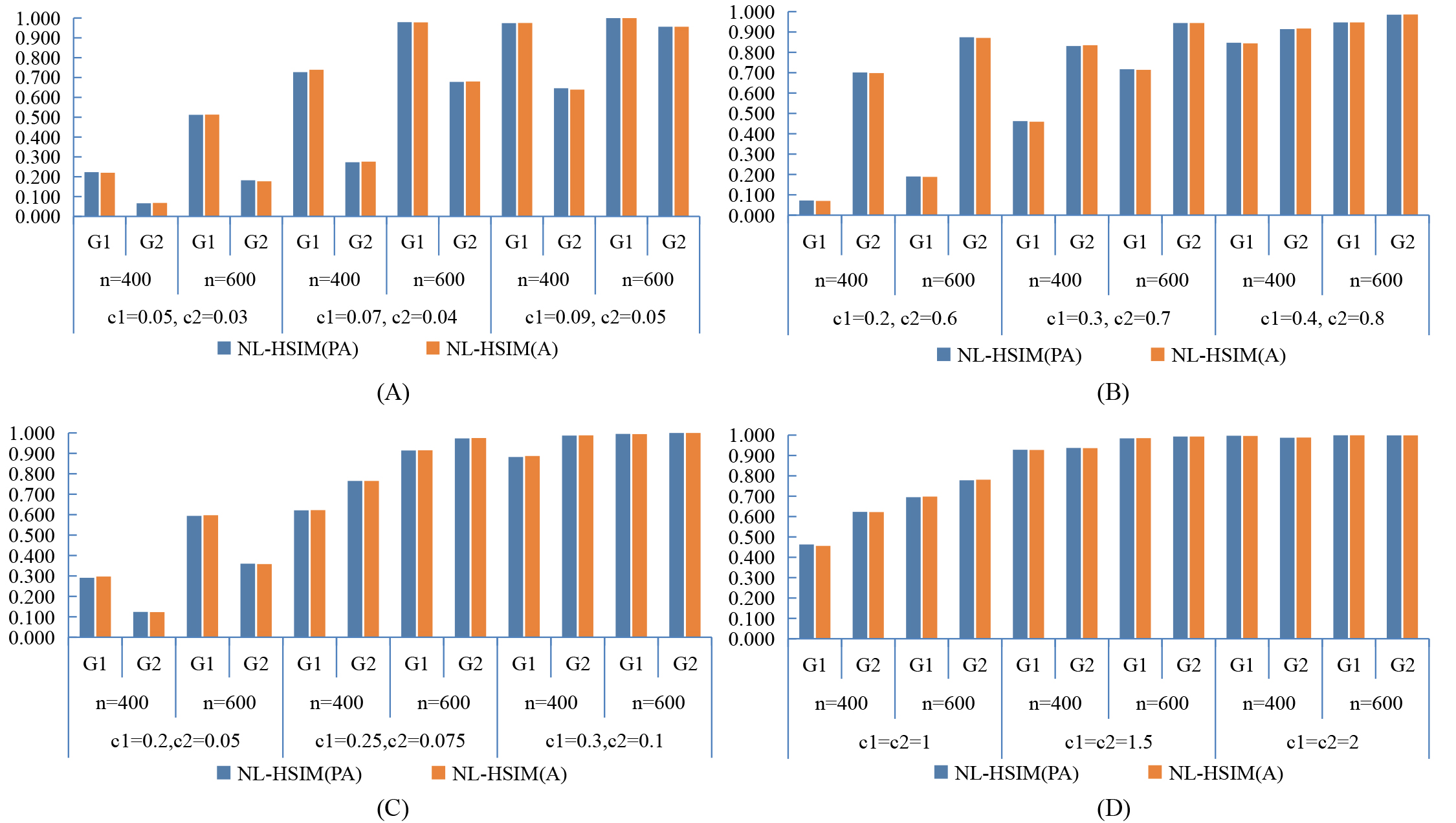


**Power comparison of NL-HSIM(A) and NL-HSIM(PA) of Case II**

Figure S9 compares NL-HSIM(A) and NL-HSIM(PA) in different simulation settings using real SNP data from chromosome 8. In the nonlinear CWSM (A) and DSSM (B) scenarios, NL-HSIM(PA) generally outperformed NL-HSIM(A), particularly in larger groups (G2), while both methods performed similarly in the linear CWSM (C) and DSSM (D) settings.

**Power comparison between NL-HSIM(O) and Linear-HSIM under linear scenarios of Case III**

Figures S10–S11 compare the empirical power of NL-HSIM(O) and Linear-HSIM under linear CWSM and DSSM settings with continuous predictors. Both methods consistently achieved high power across all scenarios, with only minor differences in specific cases. These results demonstrate that when the underlying relationship is linear, NL-HSIM(O) performs similarly to Linear-HSIM, confirming that the omnibus test retains efficiency in linear settings while still capturing nonlinear effects in more complex scenarios.

Figure S10. Power comparison of NL-HSIM(O) and Linear-HSIM under linear CWSM with continuous predictors: (A) *c*_1_=*c*_2_=0.03; (B) *c*_1_=*c*_2_=0.04; (C) *c*_1_=*c*_2_=0.05.


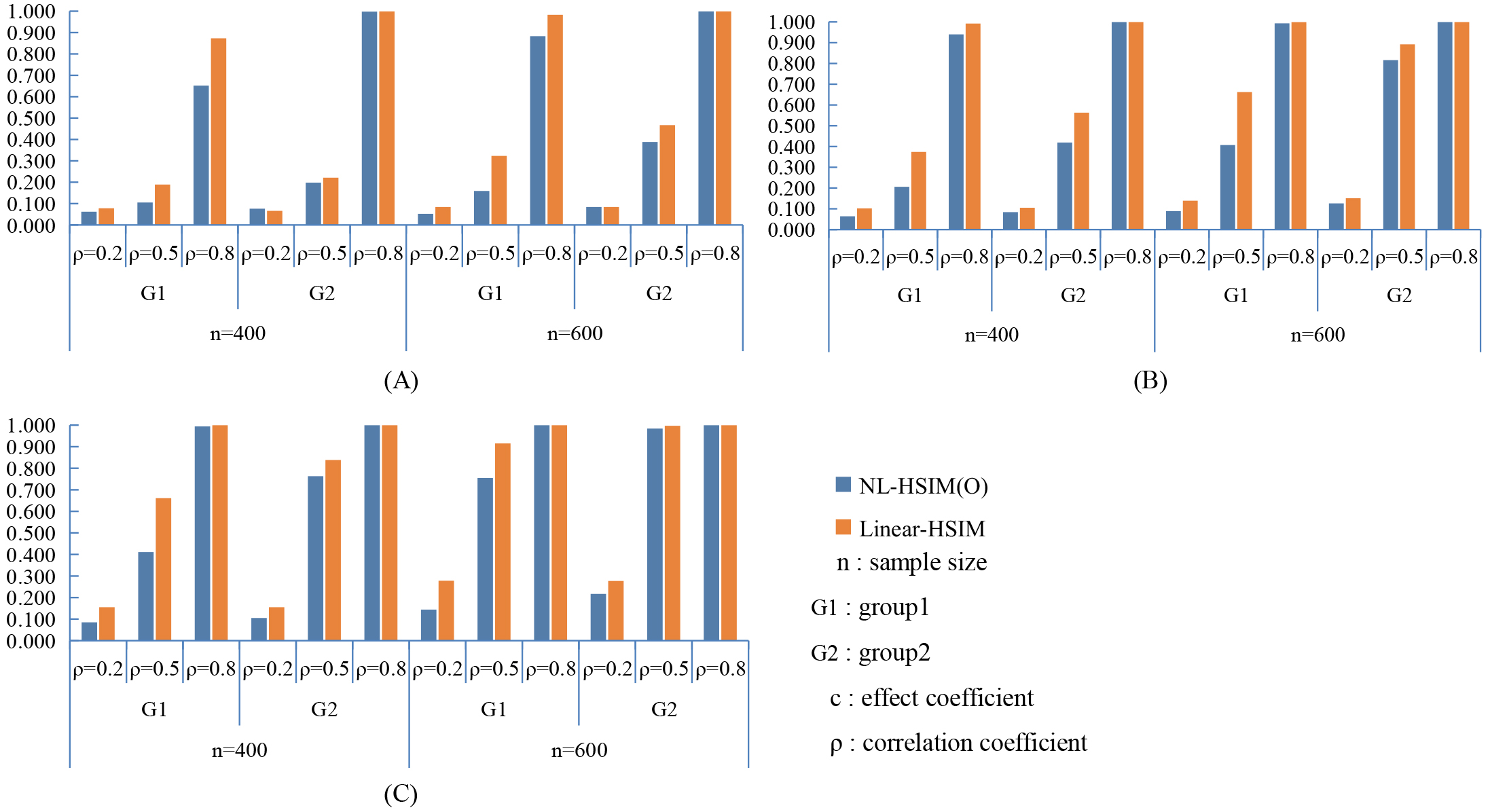


**Empirical type I error control and Power Comparison among NL-HSIM(P), NL-HSIM(A), and NL-HSIM(PA) in Case III**

Figure S12 presents the empirical type I error rates for NL-HSIM(P), NL-HSIM(A), and NL-HSIM(PA) with continuous predictors. NL-HSIM(A) and NL-HSIM(PA) maintained type I error rates close to the nominal 0.05 level across all scenarios, while NL-HSIM(P) was consistently conservative, often yielding rates below 0.05. Error control improved with larger sample sizes, supporting the validity of the framework under the global null.

Figures S13–S14 compare the empirical power of NL-HSIM(P), NL-HSIM(A), and NL-HSIM(PA) in nonlinear CWSM and DSSM scenarios with continuous predictors. Power increased steadily with effect size in all settings, with NL-HSIM(PA) generally achieving the highest power, particularly in larger groups (G2) under nonlinear DSSM. NL-HSIM(A) remained competitive under strong signals, while NL-HSIM(P) consistently lagged behind but showed significant improvement as signal strength and sample size increased.


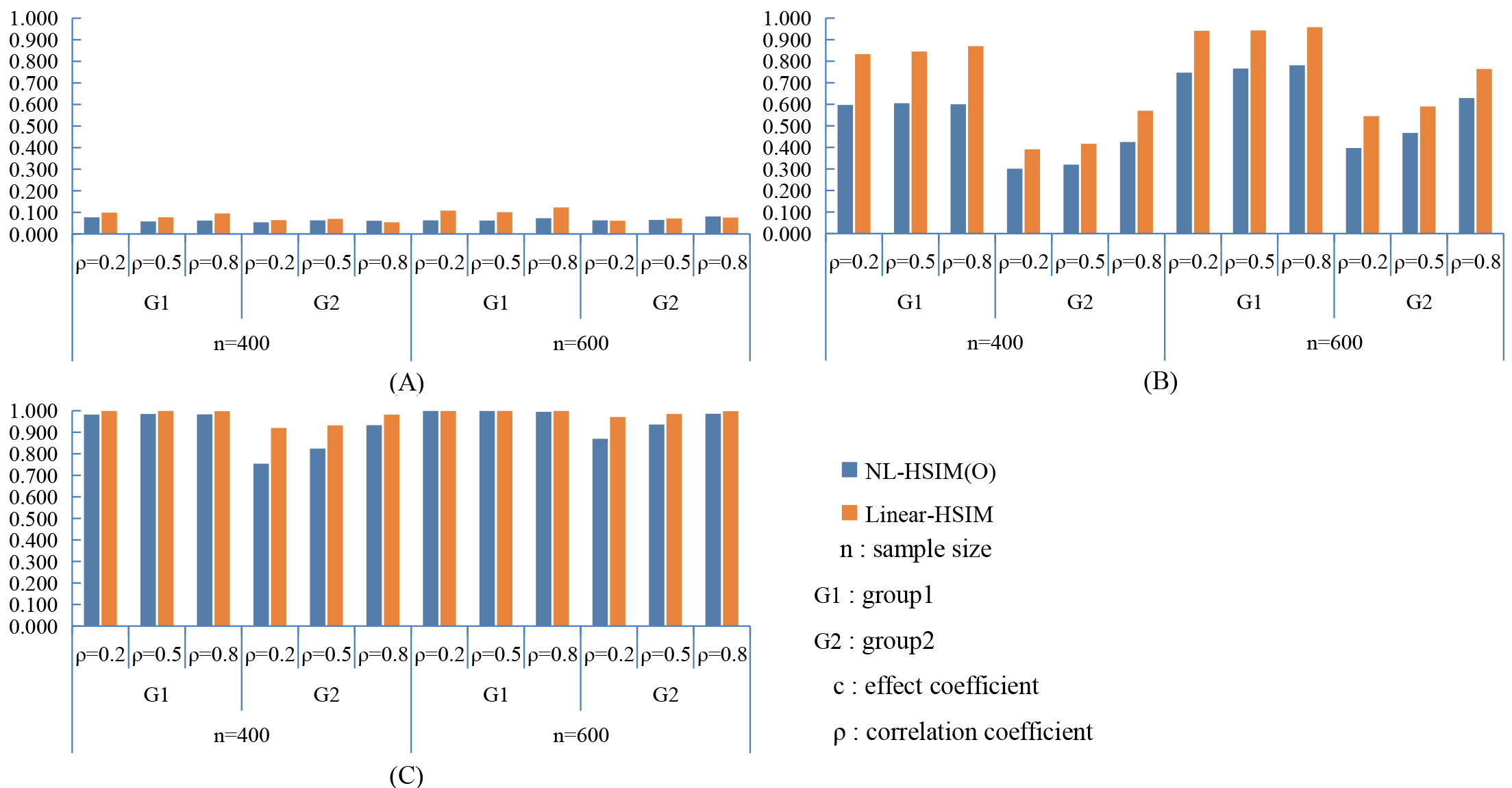


Figure S11. Power comparison of NL-HSIM(O) and Linear-HSIM under linear DSSM with continuous predictors: (A) *c*_1_=*c*_2_=0.1; (B) *c*_1_=*c*_2_=0.3; (C) *c*_1_=*c*_2_=0.5.


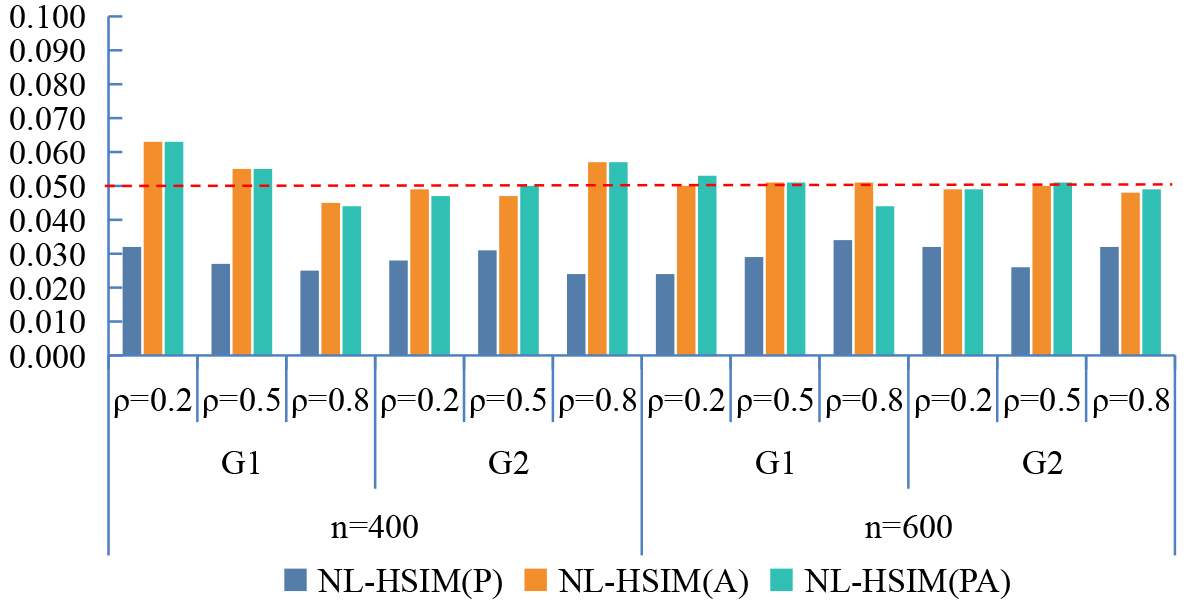


Figure S12. Empirical type I error comparison among NL-HSIM(P), NL-HSIM(A), and NL-HSIM(PA) with continuous predictors.

Figures S15 and S16 show the empirical power of NL-HSIM(P), NL-HSIM(A), and NL-HSIM(PA) in linear CWSM and DSSM scenarios with continuous predictors. Across varying effect sizes, correlations, and sample sizes, the three methods performed similarly, with only minor differences in specific cases. These results confirm that the nonlinear extensions retain their efficiency even in linear settings.


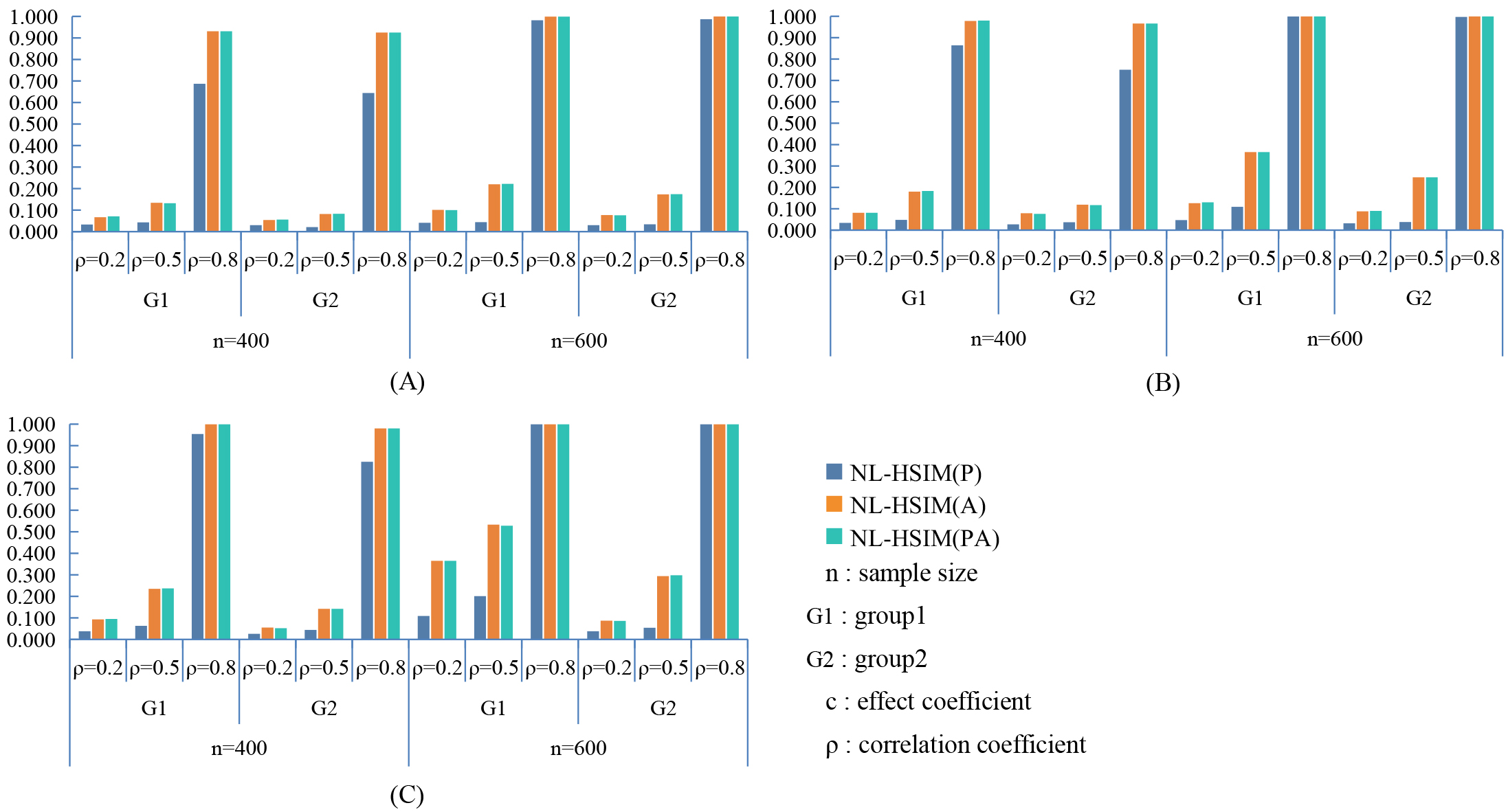


Figure S13. Empirical power comparison among NL-HSIM(P), NL-HSIM(A), and NL-HSIM(PA) in nonlinear CWSM scenarios with continuous predictors under varying effect sizes: (A) *c*_1_=0.06, *c*_2_=0.07; (B) *c*_1_=0.07, *c*_2_=0.08; (C) *c*_1_=0.08, *c*_2_=0.09.


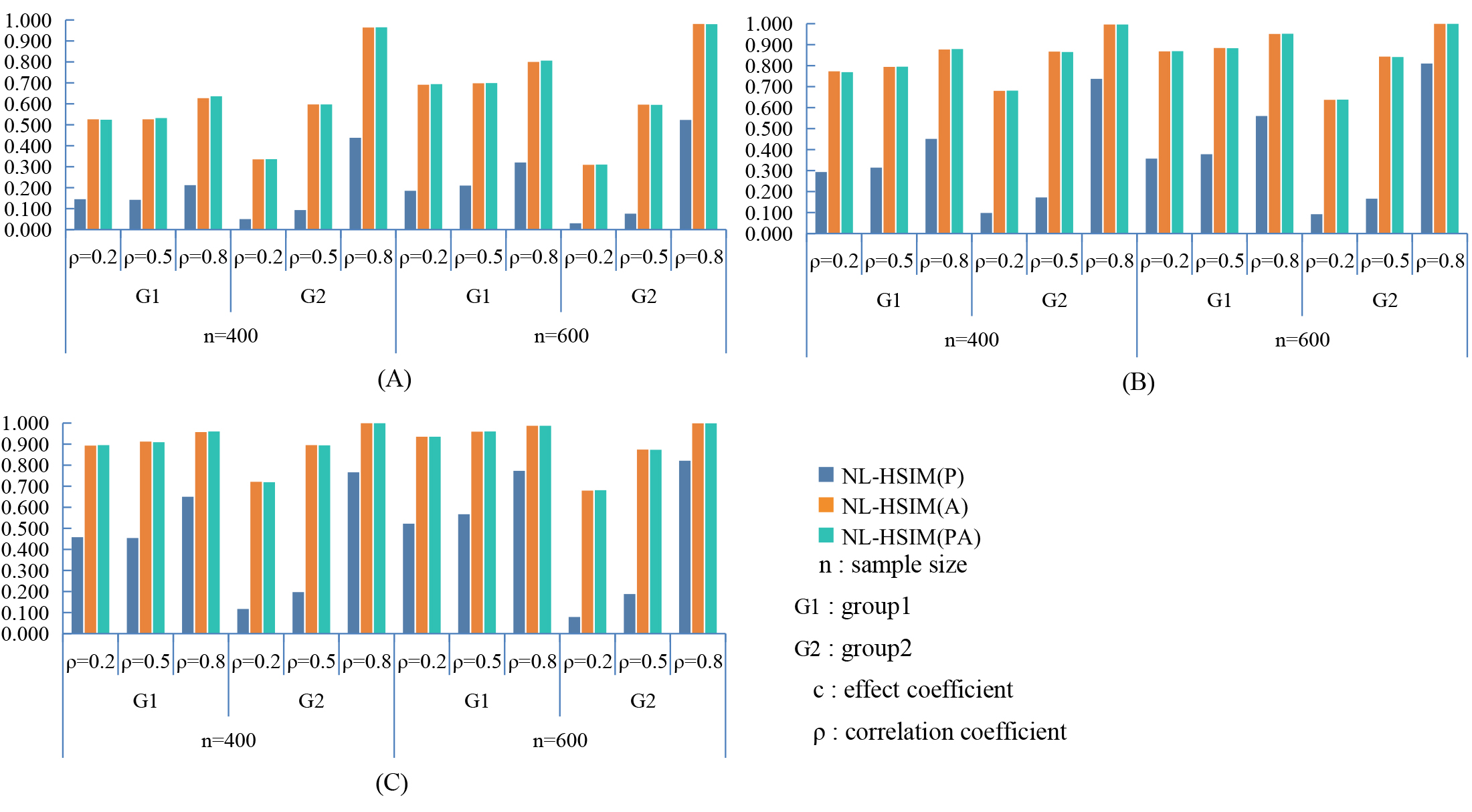


Figure S14. Empirical power comparison among NL-HSIM(P), NL-HSIM(A), and NL-HSIM(PA) in nonlinear DSSM scenarios with continuous predictors under varying effect sizes: (A) *c*_1_=0.3, *c*_2_=0.4; (B) *c*_1_=0.4, *c*_2_=1.2; (C) *c*_1_=0.5, *c*_2_=2.


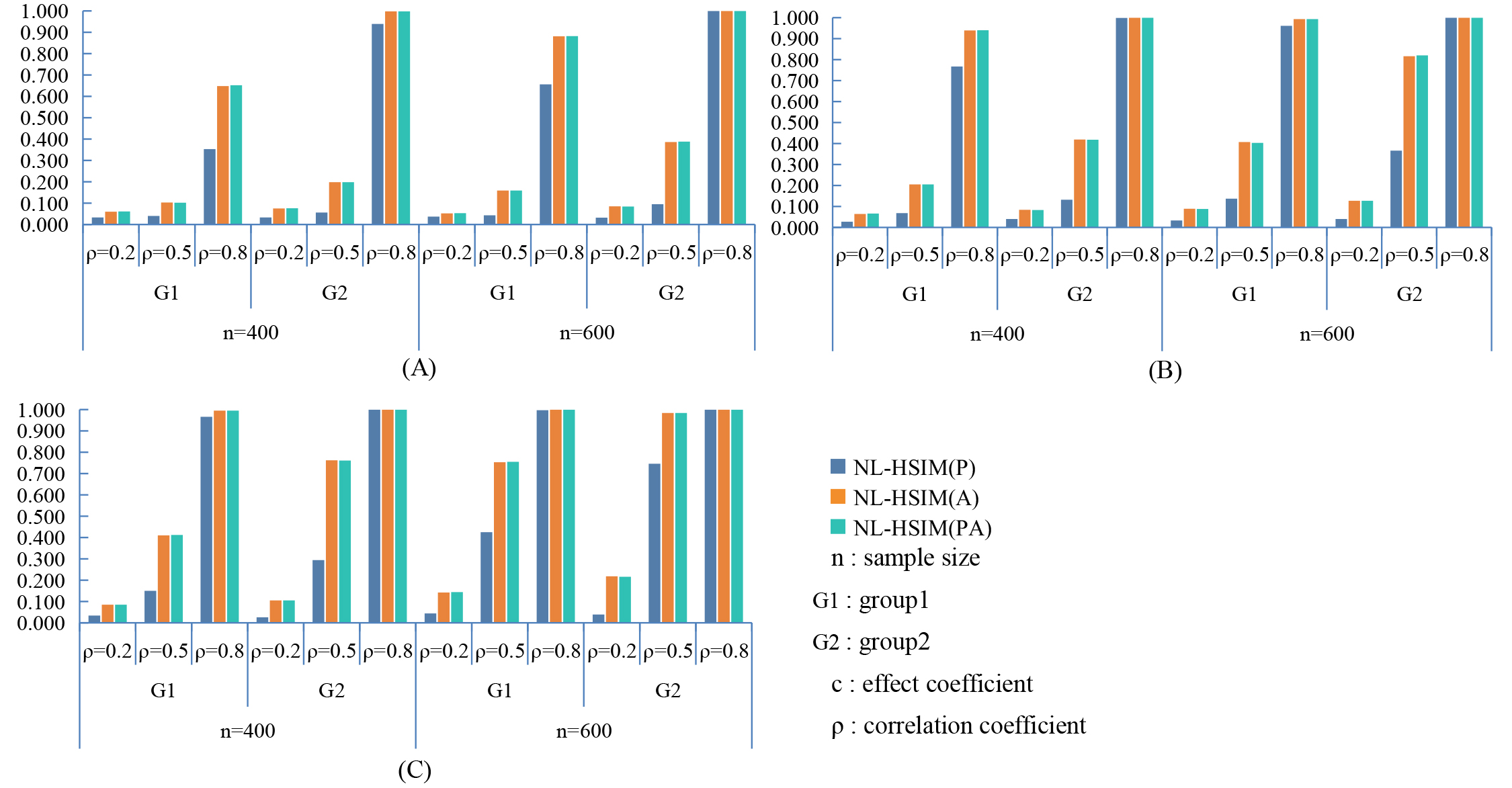


Figure S15. Power comparison among NL-HSIM(P), NL-HSIM(A), and NL-HSIM(PA) in linear CWSM scenarios with continuous predictors: (A) *c*_1_=*c*_2_=0.03; (B) *c*_1_=*c*_2_=0.04; (C) *c*_1_=*c*_2_=0.05.


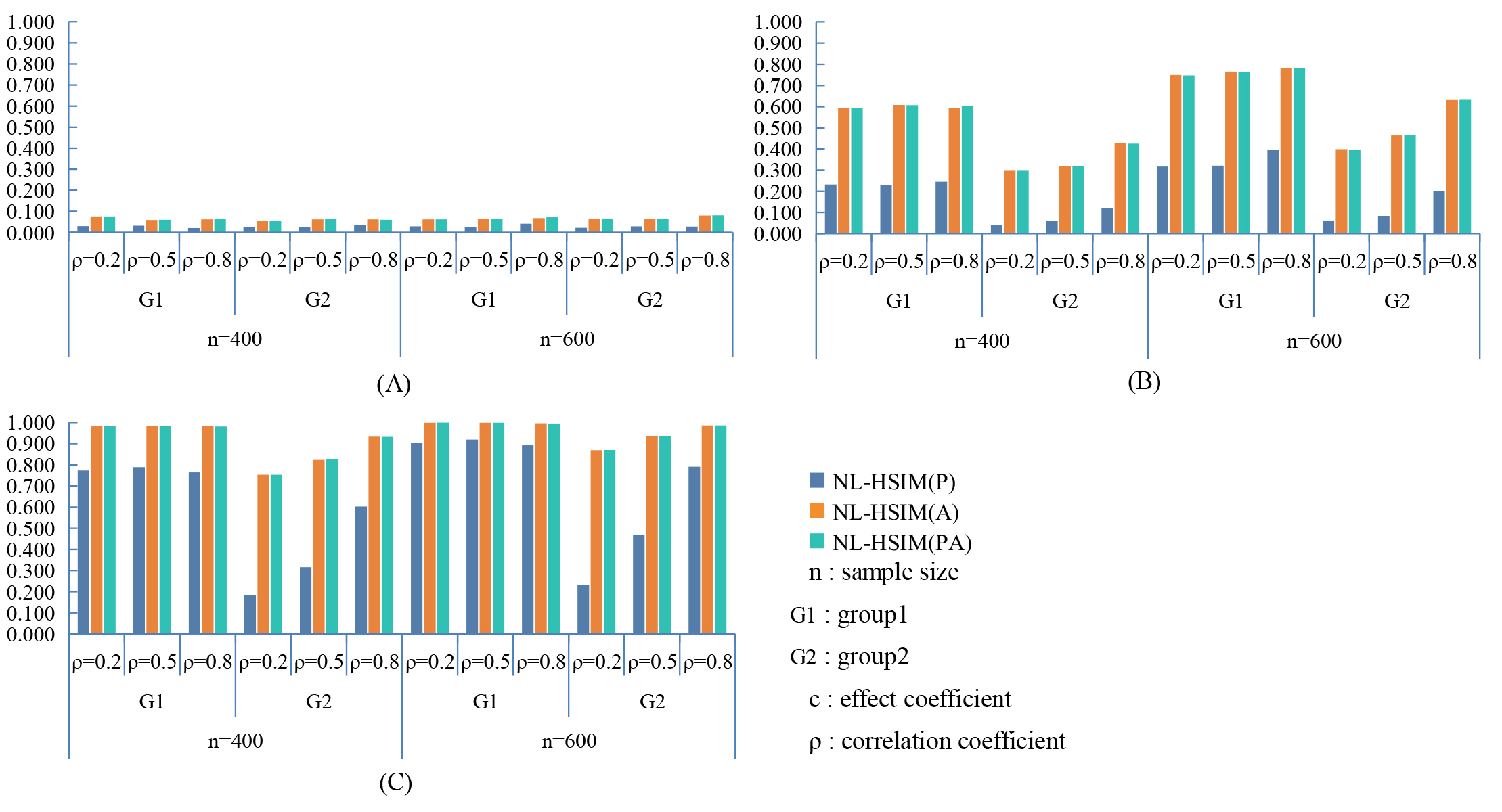


Figure S16. Power comparison among NL-HSIM(P), NL-HSIM(A), and NL-HSIM(PA) in linear DSSM scenarios with continuous predictors: (A) *c*_1_=*c*_2_=0.1; (B) *c*_1_=*c*_2_=0.3; (C) *c*_1_=*c*_2_=0.5.

The numerical results are presented in the figures in both the main text and the Supplementary File. To complement these visual summaries, additional tables (Tables S2–S17) provide detailed numerical comparisons of type I error control and power across different methods and scenarios.

| *n* | *ρ* | NL-HSIM(P) | | NL-HSIM(A) | | NL-HSIM(PA) | | NL-HSIM(O) | | Linear-HSIM | | SKAT(L) | | SKAT(IBS) | | aSPU | |
| --- | --- | --- | --- | --- | --- | --- | --- | --- | --- | --- | --- | --- | --- | --- | --- | --- | --- |
|  |  | Group1 | Group2 | Group1 | Group2 | Group1 | Group2 | Group1 | Group2 | Group1 | Group2 | Group1 | Group2 | Group1 | Group2 | Group1 | Group2 |
| 400 | 0.2 | 0.027 | 0.035 | 0.056 | 0.049 | 0.056 | 0.051 | 0.057 | 0.050 | 0.064 | 0.062 | 0.043 | 0.015 | 0.032 | 0.008 | 0.051 | 0.027 |
|  | 0.5 | 0.032 | 0.033 | 0.047 | 0.053 | 0.049 | 0.053 | 0.047 | 0.054 | 0.049 | 0.062 | 0.045 | 0.020 | 0.044 | 0.010 | 0.038 | 0.044 |
|  | 0.8 | 0.036 | 0.025 | 0.049 | 0.047 | 0.052 | 0.044 | 0.052 | 0.045 | 0.053 | 0.055 | 0.042 | 0.028 | 0.040 | 0.028 | 0.042 | 0.038 |
| 600 | 0.2 | 0.027 | 0.025 | 0.051 | 0.053 | 0.051 | 0.052 | 0.050 | 0.053 | 0.051 | 0.055 | 0.038 | 0.026 | 0.042 | 0.015 | 0.034 | 0.028 |
|  | 0.5 | 0.023 | 0.024 | 0.069 | 0.052 | 0.068 | 0.052 | 0.068 | 0.052 | 0.050 | 0.066 | 0.052 | 0.03 | 0.051 | 0.018 | 0.040 | 0.035 |
|  | 0.8 | 0.030 | 0.028 | 0.046 | 0.063 | 0.050 | 0.064 | 0.049 | 0.064 | 0.058 | 0.065 | 0.052 | 0.047 | 0.052 | 0.035 | 0.036 | 0.044 |

Table S2. Type I error comparison among methods in Case I.

Table S3. Power comparison among methods under the nonlinear CWSM model in Case I.

| *c* | *n* | *ρ* | NL-HSIM(P) | | NL-HSIM(A) | | NL-HSIM(PA) | | NL-HSIM(O) | | Linear-HSIM | | SKAT(L) | | SKAT(IBS) | | aSPU | |
| --- | --- | --- | --- | --- | --- | --- | --- | --- | --- | --- | --- | --- | --- | --- | --- | --- | --- | --- |
|  |  |  | Group1 | Group2 | Group1 | Group2 | Group1 | Group2 | Group1 | Group2 | Group1 | Group2 | Group1 | Group2 | Group1 | Group2 | Group1 | Group2 |
| *c*_1_=0.1  *c*_2_=0.2 | 400 | 0.2 | 0.042 | 0.033 | 0.080 | 0.111 | 0.084 | 0.109 | 0.080 | 0.110 | 0.064 | 0.055 | 0.048 | 0.016 | 0.133 | 0.146 | 0.042 | 0.035 |
|  |  | 0.5 | 0.042 | 0.055 | 0.104 | 0.144 | 0.107 | 0.145 | 0.109 | 0.146 | 0.055 | 0.068 | 0.041 | 0.017 | 0.156 | 0.210 | 0.041 | 0.042 |
|  |  | 0.8 | 0.105 | 0.389 | 0.413 | 0.716 | 0.417 | 0.712 | 0.417 | 0.711 | 0.049 | 0.053 | 0.047 | 0.039 | 0.375 | 0.551 | 0.047 | 0.050 |
|  | 600 | 0.2 | 0.027 | 0.092 | 0.090 | 0.288 | 0.089 | 0.289 | 0.089 | 0.290 | 0.053 | 0.062 | 0.043 | 0.015 | 0.240 | 0.464 | 0.028 | 0.037 |
|  |  | 0.5 | 0.033 | 0.187 | 0.132 | 0.447 | 0.129 | 0.450 | 0.130 | 0.448 | 0.040 | 0.067 | 0.041 | 0.023 | 0.283 | 0.568 | 0.034 | 0.037 |
|  |  | 0.8 | 0.291 | 0.881 | 0.774 | 0.990 | 0.773 | 0.990 | 0.774 | 0.991 | 0.062 | 0.052 | 0.040 | 0.029 | 0.590 | 0.840 | 0.036 | 0.044 |
| *c*_1_=0.2  *c*_2_=0.4 | 400 | 0.2 | 0.060 | 0.071 | 0.293 | 0.218 | 0.290 | 0.215 | 0.292 | 0.217 | 0.066 | 0.040 | 0.045 | 0.018 | 0.576 | 0.320 | 0.046 | 0.037 |
|  |  | 0.5 | 0.095 | 0.099 | 0.395 | 0.290 | 0.395 | 0.293 | 0.396 | 0.291 | 0.059 | 0.045 | 0.043 | 0.019 | 0.648 | 0.385 | 0.037 | 0.035 |
|  |  | 0.8 | 0.828 | 0.612 | 0.992 | 0.860 | 0.992 | 0.861 | 0.992 | 0.862 | 0.050 | 0.059 | 0.041 | 0.029 | 0.795 | 0.687 | 0.042 | 0.043 |
|  | 600 | 0.2 | 0.218 | 0.342 | 0.610 | 0.615 | 0.610 | 0.613 | 0.611 | 0.614 | 0.072 | 0.060 | 0.045 | 0.018 | 0.852 | 0.740 | 0.038 | 0.033 |
|  |  | 0.5 | 0.313 | 0.532 | 0.851 | 0.785 | 0.850 | 0.784 | 0.850 | 0.784 | 0.056 | 0.056 | 0.042 | 0.025 | 0.843 | 0.783 | 0.032 | 0.036 |
|  |  | 0.8 | 0.999 | 0.981 | 1.000 | 1.000 | 1.000 | 1.000 | 1.000 | 1.000 | 0.057 | 0.057 | 0.046 | 0.035 | 0.877 | 0.905 | 0.036 | 0.052 |
| *c*_1_=0.3  *c*_2_=0.6 | 400 | 0.2 | 0.218 | 0.084 | 0.654 | 0.255 | 0.652 | 0.258 | 0.654 | 0.258 | 0.057 | 0.050 | 0.041 | 0.018 | 0.834 | 0.370 | 0.045 | 0.037 |
|  |  | 0.5 | 0.349 | 0.118 | 0.816 | 0.350 | 0.816 | 0.348 | 0.817 | 0.350 | 0.066 | 0.055 | 0.040 | 0.018 | 0.854 | 0.430 | 0.041 | 0.029 |
|  |  | 0.8 | 0.993 | 0.671 | 1.000 | 0.899 | 1.000 | 0.900 | 1.000 | 0.899 | 0.054 | 0.051 | 0.040 | 0.027 | 0.861 | 0.702 | 0.035 | 0.041 |
|  | 600 | 0.2 | 0.790 | 0.453 | 0.970 | 0.718 | 0.968 | 0.718 | 0.970 | 0.719 | 0.062 | 0.056 | 0.041 | 0.016 | 0.964 | 0.790 | 0.038 | 0.032 |
|  |  | 0.5 | 0.882 | 0.625 | 0.997 | 0.848 | 0.997 | 0.848 | 0.997 | 0.848 | 0.056 | 0.051 | 0.045 | 0.025 | 0.941 | 0.828 | 0.035 | 0.029 |
|  |  | 0.8 | 1.000 | 0.991 | 1.000 | 1.000 | 1.000 | 1.000 | 1.000 | 1.000 | 0.060 | 0.067 | 0.035 | 0.037 | 0.928 | 0.918 | 0.031 | 0.051 |

Table S4. Power comparison among methods under the nonlinear DSSM model in Case I.

| *c* | *n* | *ρ* | NL-HSIM(P) | | NL-HSIM(A) | | NL-HSIM(PA) | | NL-HSIM(O) | | Linear-HSIM | | SKAT(L) | | SKAT(IBS) | | aSPU | |
| --- | --- | --- | --- | --- | --- | --- | --- | --- | --- | --- | --- | --- | --- | --- | --- | --- | --- | --- |
|  |  |  | Group1 | Group2 | Group1 | Group2 | Group1 | Group2 | Group1 | Group2 | Group1 | Group2 | Group1 | Group2 | Group1 | Group2 | Group1 | Group2 |
| *c*_1_=0.5  *c*_2_=1 | 400 | 0.2 | 0.298 | 0.286 | 0.665 | 0.653 | 0.664 | 0.654 | 0.668 | 0.652 | 0.067 | 0.045 | 0.037 | 0.018 | 0.306 | 0.277 | 0.041 | 0.037 |
|  |  | 0.5 | 0.293 | 0.299 | 0.648 | 0.691 | 0.648 | 0.693 | 0.648 | 0.693 | 0.049 | 0.066 | 0.050 | 0.013 | 0.271 | 0.330 | 0.034 | 0.027 |
|  |  | 0.8 | 0.396 | 0.362 | 0.695 | 0.865 | 0.684 | 0.862 | 0.690 | 0.861 | 0.052 | 0.048 | 0.041 | 0.013 | 0.187 | 0.330 | 0.037 | 0.027 |
|  | 600 | 0.2 | 0.238 | 0.271 | 0.606 | 0.622 | 0.601 | 0.623 | 0.606 | 0.622 | 0.057 | 0.068 | 0.044 | 0.023 | 0.487 | 0.538 | 0.029 | 0.034 |
|  |  | 0.5 | 0.258 | 0.293 | 0.650 | 0.672 | 0.649 | 0.672 | 0.647 | 0.674 | 0.061 | 0.057 | 0.042 | 0.026 | 0.425 | 0.556 | 0.038 | 0.027 |
|  |  | 0.8 | 0.374 | 0.346 | 0.741 | 0.869 | 0.738 | 0.868 | 0.743 | 0.867 | 0.061 | 0.051 | 0.041 | 0.027 | 0.333 | 0.544 | 0.030 | 0.042 |
| *c*_1_=1  *c*_2_=3 | 400 | 0.2 | 0.675 | 0.473 | 0.915 | 0.818 | 0.914 | 0.813 | 0.915 | 0.818 | 0.064 | 0.051 | 0.037 | 0.012 | 0.497 | 0.413 | 0.033 | 0.030 |
|  |  | 0.5 | 0.663 | 0.509 | 0.921 | 0.864 | 0.921 | 0.865 | 0.921 | 0.866 | 0.053 | 0.070 | 0.045 | 0.021 | 0.493 | 0.443 | 0.036 | 0.027 |
|  |  | 0.8 | 0.743 | 0.518 | 0.940 | 0.942 | 0.940 | 0.942 | 0.941 | 0.940 | 0.044 | 0.063 | 0.034 | 0.022 | 0.526 | 0.517 | 0.036 | 0.035 |
|  | 600 | 0.2 | 0.583 | 0.467 | 0.922 | 0.790 | 0.922 | 0.795 | 0.922 | 0.795 | 0.071 | 0.053 | 0.040 | 0.015 | 0.515 | 0.639 | 0.038 | 0.036 |
|  |  | 0.5 | 0.597 | 0.440 | 0.929 | 0.831 | 0.929 | 0.833 | 0.928 | 0.831 | 0.061 | 0.047 | 0.042 | 0.022 | 0.471 | 0.631 | 0.035 | 0.028 |
|  |  | 0.8 | 0.765 | 0.502 | 0.971 | 0.951 | 0.971 | 0.949 | 0.971 | 0.950 | 0.049 | 0.057 | 0.038 | 0.028 | 0.496 | 0.608 | 0.028 | 0.037 |
| *c*_1_=1.5  *c*_2_=5 | 400 | 0.2 | 0.808 | 0.481 | 0.978 | 0.818 | 0.975 | 0.817 | 0.977 | 0.818 | 0.062 | 0.049 | 0.032 | 0.013 | 0.496 | 0.429 | 0.027 | 0.025 |
|  |  | 0.5 | 0.794 | 0.525 | 0.967 | 0.846 | 0.966 | 0.85 | 0.967 | 0.846 | 0.054 | 0.055 | 0.037 | 0.021 | 0.497 | 0.451 | 0.037 | 0.028 |
|  |  | 0.8 | 0.880 | 0.55 | 0.987 | 0.948 | 0.987 | 0.944 | 0.987 | 0.945 | 0.047 | 0.06 | 0.041 | 0.022 | 0.530 | 0.523 | 0.031 | 0.038 |
|  | 600 | 0.2 | 0.760 | 0.482 | 0.978 | 0.814 | 0.978 | 0.812 | 0.978 | 0.814 | 0.074 | 0.052 | 0.052 | 0.015 | 0.513 | 0.640 | 0.041 | 0.033 |
|  |  | 0.5 | 0.754 | 0.469 | 0.978 | 0.84 | 0.979 | 0.839 | 0.979 | 0.838 | 0.052 | 0.055 | 0.039 | 0.021 | 0.466 | 0.641 | 0.036 | 0.032 |
|  |  | 0.8 | 0.883 | 0.533 | 0.996 | 0.965 | 0.996 | 0.965 | 0.995 | 0.964 | 0.059 | 0.051 | 0.041 | 0.027 | 0.495 | 0.611 | 0.036 | 0.035 |

Table S5. Power comparison among methods under the linear CWSM model in Case I.

| *c* | *n* | *ρ* | NL-HSIM(P) | | NL-HSIM(A) | | NL-HSIM(PA) | | NL-HSIM(O) | | Linear-HSIM | | SKAT(L) | | SKAT(IBS) | | | aSPU | |
| --- | --- | --- | --- | --- | --- | --- | --- | --- | --- | --- | --- | --- | --- | --- | --- | --- | --- | --- | --- |
|  |  |  | Group1 | Group2 | Group1 | Group2 | Group1 | Group2 | Group1 | Group2 | Group1 | Group2 | Group1 | Group2 | Group1 | Group2 | Group1 | | Group2 |
| *c*_1_=0.05  *c*_2_=0.05 | 400 | 0.2 | 0.035 | 0.033 | 0.076 | 0.096 | 0.077 | 0.095 | 0.077 | 0.095 | 0.115 | 0.092 | 0.170 | 0.115 | 0.158 | 0.064 | 0.294 | | 0.292 |
|  |  | 0.5 | 0.072 | 0.088 | 0.195 | 0.359 | 0.196 | 0.359 | 0.195 | 0.358 | 0.305 | 0.420 | 0.549 | 0.648 | 0.570 | 0.573 | 0.707 | | 0.82 |
|  |  | 0.8 | 0.809 | 0.998 | 0.948 | 1.000 | 0.948 | 1.000 | 0.948 | 1.000 | 0.977 | 1.000 | 0.932 | 0.999 | 0.911 | 0.995 | 1.000 | | 1.000 |
|  | 600 | 0.2 | 0.030 | 0.032 | 0.084 | 0.113 | 0.079 | 0.115 | 0.080 | 0.114 | 0.157 | 0.150 | 0.307 | 0.280 | 0.311 | 0.216 | 0.473 | | 0.528 |
|  |  | 0.5 | 0.107 | 0.272 | 0.350 | 0.723 | 0.352 | 0.724 | 0.350 | 0.721 | 0.570 | 0.769 | 0.774 | 0.917 | 0.779 | 0.899 | 0.939 | | 0.994 |
|  |  | 0.8 | 0.981 | 1.000 | 0.996 | 1.000 | 0.996 | 1.000 | 0.996 | 1.000 | 0.999 | 1.000 | 0.952 | 0.999 | 0.941 | 0.998 | 1.000 | | 1.000 |
| *c*_1_=0.075  *c*_2_=0.075 | 400 | 0.2 | 0.037 | 0.038 | 0.127 | 0.193 | 0.129 | 0.194 | 0.129 | 0.194 | 0.215 | 0.217 | 0.423 | 0.365 | 0.428 | 0.278 | 0.672 | | 0.647 |
|  |  | 0.5 | 0.320 | 0.444 | 0.645 | 0.869 | 0.644 | 0.869 | 0.644 | 0.869 | 0.816 | 0.922 | 0.890 | 0.964 | 0.890 | 0.947 | 0.986 | | 0.999 |
|  |  | 0.8 | 1.000 | 1.000 | 1.000 | 1.000 | 1.000 | 1.000 | 1.000 | 1.000 | 1.000 | 1.000 | 0.983 | 1.000 | 0.974 | 0.999 | 1.000 | | 1.000 |
|  | 600 | 0.2 | 0.069 | 0.058 | 0.229 | 0.350 | 0.230 | 0.348 | 0.230 | 0.349 | 0.411 | 0.429 | 0.670 | 0.728 | 0.685 | 0.668 | 0.899 | | 0.946 |
|  |  | 0.5 | 0.718 | 0.882 | 0.926 | 0.996 | 0.927 | 0.996 | 0.926 | 0.996 | 0.976 | 1.000 | 0.964 | 0.997 | 0.959 | 0.996 | 1.000 | | 1.000 |
|  |  | 0.8 | 1.000 | 1.000 | 1.000 | 1.000 | 1.000 | 1.000 | 1.000 | 1.000 | 1.000 | 1.000 | 0.983 | 1.000 | 0.976 | 0.999 | 1.000 | | 1.000 |
| *c*_1_=0.1  *c*_2_=0.1 | 400 | 0.2 | 0.069 | 0.067 | 0.296 | 0.359 | 0.291 | 0.355 | 0.294 | 0.357 | 0.478 | 0.437 | 0.983 | 0.700 | 0.976 | 0.621 | 1.000 | | 0.939 |
|  |  | 0.5 | 0.781 | 0.818 | 0.958 | 0.991 | 0.958 | 0.989 | 0.957 | 0.990 | 0.984 | 0.997 | 0.964 | 0.995 | 0.967 | 0.996 | 1.000 | | 1.000 |
|  |  | 0.8 | 1.000 | 1.000 | 1.000 | 1.000 | 1.000 | 1.000 | 1.000 | 1.000 | 1.000 | 1.000 | 0.991 | 1.000 | 0.990 | 1.000 | 1.000 | | 1.000 |
|  | 600 | 0.2 | 0.234 | 0.175 | 0.587 | 0.719 | 0.589 | 0.722 | 0.588 | 0.724 | 0.781 | 0.840 | 0.892 | 0.949 | 0.908 | 0.942 | 0.995 | | 1.000 |
|  |  | 0.5 | 0.983 | 0.998 | 0.999 | 1.000 | 0.999 | 1.000 | 0.999 | 1.000 | 1.000 | 1.000 | 0.989 | 1.000 | 0.987 | 1.000 | 1.000 | | 1.000 |
|  |  | 0.8 | 1.000 | 1.000 | 1.000 | 1.000 | 1.000 | 1.000 | 1.000 | 1.000 | 1.000 | 1.000 | 0.986 | 1.000 | 0.982 | 1.000 | 1.000 | | 1.000 |

Table S6. Power comparison among methods under the linear DSSM model in Case I.

| *c* | *n* | *ρ* | NL-HSIM(P) | | NL-HSIM(A) | | NL-HSIM(PA) | | NL-HSIM(O) | | Linear-HSIM | | SKAT(L) | | SKAT(IBS) | | aSPU | |
| --- | --- | --- | --- | --- | --- | --- | --- | --- | --- | --- | --- | --- | --- | --- | --- | --- | --- | --- |
|  |  |  | Group1 | Group2 | Group1 | Group2 | Group1 | Group2 | Group1 | Group2 | Group1 | Group2 | Group1 | Group2 | Group1 | Group2 | Group1 | Group2 |
| *c*_1_=0.1  *c*_2_=0.05 | 400 | 0.2 | 0.033 | 0.038 | 0.067 | 0.054 | 0.063 | 0.051 | 0.066 | 0.053 | 0.089 | 0.059 | 0.060 | 0.016 | 0.046 | 0.007 | 0.081 | 0.027 |
|  |  | 0.5 | 0.030 | 0.034 | 0.048 | 0.049 | 0.046 | 0.050 | 0.047 | 0.050 | 0.071 | 0.060 | 0.056 | 0.018 | 0.052 | 0.011 | 0.065 | 0.048 |
|  |  | 0.8 | 0.033 | 0.041 | 0.062 | 0.068 | 0.061 | 0.067 | 0.062 | 0.068 | 0.074 | 0.075 | 0.056 | 0.029 | 0.061 | 0.027 | 0.077 | 0.039 |
|  | 600 | 0.2 | 0.028 | 0.027 | 0.059 | 0.057 | 0.058 | 0.055 | 0.058 | 0.055 | 0.077 | 0.059 | 0.062 | 0.033 | 0.061 | 0.016 | 0.078 | 0.045 |
|  |  | 0.5 | 0.034 | 0.038 | 0.077 | 0.060 | 0.075 | 0.061 | 0.076 | 0.060 | 0.075 | 0.069 | 0.071 | 0.029 | 0.065 | 0.021 | 0.087 | 0.029 |
|  |  | 0.8 | 0.036 | 0.033 | 0.069 | 0.082 | 0.066 | 0.079 | 0.067 | 0.080 | 0.084 | 0.099 | 0.087 | 0.049 | 0.076 | 0.037 | 0.098 | 0.047 |
| *c*_1_=0.3  *c*_2_=0.15 | 400 | 0.2 | 0.157 | 0.041 | 0.461 | 0.124 | 0.458 | 0.124 | 0.461 | 0.125 | 0.655 | 0.165 | 0.253 | 0.023 | 0.284 | 0.006 | 0.851 | 0.068 |
|  |  | 0.5 | 0.162 | 0.130 | 0.488 | 0.404 | 0.488 | 0.404 | 0.488 | 0.406 | 0.650 | 0.432 | 0.264 | 0.021 | 0.295 | 0.013 | 0.877 | 0.069 |
|  |  | 0.8 | 0.190 | 0.370 | 0.469 | 0.749 | 0.469 | 0.748 | 0.470 | 0.748 | 0.653 | 0.781 | 0.379 | 0.043 | 0.386 | 0.032 | 0.888 | 0.073 |
|  | 600 | 0.2 | 0.208 | 0.058 | 0.604 | 0.251 | 0.609 | 0.251 | 0.607 | 0.253 | 0.803 | 0.283 | 0.386 | 0.038 | 0.415 | 0.020 | 0.982 | 0.136 |
|  |  | 0.5 | 0.214 | 0.262 | 0.616 | 0.626 | 0.615 | 0.626 | 0.615 | 0.628 | 0.808 | 0.657 | 0.393 | 0.044 | 0.406 | 0.033 | 0.988 | 0.124 |
|  |  | 0.8 | 0.303 | 0.628 | 0.639 | 0.910 | 0.646 | 0.910 | 0.642 | 0.910 | 0.811 | 0.941 | 0.517 | 0.065 | 0.510 | 0.058 | 0.990 | 0.151 |
| *c*_1_=0.5  *c*_2_=0.25 | 400 | 0.2 | 0.657 | 0.157 | 0.943 | 0.622 | 0.941 | 0.622 | 0.941 | 0.622 | 0.983 | 0.718 | 0.432 | 0.039 | 0.498 | 0.016 | 1.000 | 0.421 |
|  |  | 0.5 | 0.672 | 0.709 | 0.949 | 0.957 | 0.951 | 0.957 | 0.950 | 0.957 | 0.988 | 0.980 | 0.502 | 0.048 | 0.537 | 0.028 | 0.999 | 0.422 |
|  |  | 0.8 | 0.681 | 0.958 | 0.948 | 0.999 | 0.951 | 0.999 | 0.949 | 0.999 | 0.986 | 1.000 | 0.670 | 0.067 | 0.655 | 0.051 | 1.000 | 0.456 |
|  | 600 | 0.2 | 0.812 | 0.269 | 0.988 | 0.841 | 0.988 | 0.841 | 0.989 | 0.841 | 0.999 | 0.901 | 0.501 | 0.078 | 0.514 | 0.051 | 1.000 | 0.796 |
|  |  | 0.5 | 0.832 | 0.881 | 0.986 | 0.996 | 0.986 | 0.996 | 0.986 | 0.996 | 0.996 | 0.997 | 0.556 | 0.107 | 0.548 | 0.073 | 1.000 | 0.762 |
|  |  | 0.8 | 0.826 | 0.999 | 0.989 | 1.000 | 0.989 | 1.000 | 0.990 | 1.000 | 0.999 | 1.000 | 0.709 | 0.135 | 0.689 | 0.124 | 1.000 | 0.772 |

Table S7. Power comparison among methods under the nonlinear CWSM model in Case II.

| *n* | *c* | NL-HSIM(PA) | | NL-HSIM(A) | | NL-HSIM(O) | | SKAT(L) | | SKAT(IBS) | | aSPU | |
| --- | --- | --- | --- | --- | --- | --- | --- | --- | --- | --- | --- | --- | --- |
|  |  | Group1 | Group2 | Group1 | Group2 | Group1 | Group2 | Group1 | Group2 | Group1 | Group2 | Group1 | Group2 |
| 400 | *c*_1_=0.05  *c*_2_=0.03 | 0.223 | 0.066 | 0.22 | 0.068 | 0.22 | 0.065 | 0.001 | 0.001 | 0.002 | 0.000 | 0.001 | 0.003 |
| 400 | *c*_1_=0.07  *c*_2_=0.04 | 0.727 | 0.273 | 0.739 | 0.276 | 0.742 | 0.275 | 0.001 | 0.003 | 0.022 | 0.004 | 0.018 | 0.018 |
| 400 | *c*_1_=0.09  *c*_2_=0.05 | 0.974 | 0.646 | 0.975 | 0.639 | 0.979 | 0.643 | 0.001 | 0.005 | 0.161 | 0.042 | 0.086 | 0.057 |
| 600 | *c*_1_=0.05  *c*_2_=0.03 | 0.512 | 0.182 | 0.513 | 0.177 | 0.512 | 0.177 | 0.001 | 0.000 | 0.014 | 0.012 | 0.011 | 0.007 |
| 600 | *c*_1_=0.07  *c*_2_=0.04 | 0.979 | 0.678 | 0.978 | 0.680 | 0.980 | 0.681 | 0.001 | 0.003 | 0.187 | 0.112 | 0.085 | 0.067 |
| 600 | *c*_1_=0.09  *c*_2_=0.05 | 1.000 | 0.956 | 1.000 | 0.956 | 1.000 | 0.959 | 0.001 | 0.010 | 0.699 | 0.499 | 0.295 | 0.231 |

Table S8. Power comparison among methods under the nonlinear DSSM model in Case II.

| *n* | *c* | NL-HSIM(PA) | | NL-HSIM(A) | | NL-HSIM(O) | | SKAT(L) | | SKAT(IBS) | | aSPU | |
| --- | --- | --- | --- | --- | --- | --- | --- | --- | --- | --- | --- | --- | --- |
|  |  | Group1 | Group2 | Group1 | Group2 | Group1 | Group2 | Group1 | Group2 | Group1 | Group2 | Group1 | Group2 |
| 400 | *c*_1_=0.2  *c*_2_=0.6 | 0.072 | 0.701 | 0.070 | 0.698 | 0.072 | 0.706 | 0.000 | 0.000 | 0.000 | 0.000 | 0.005 | 0.966 |
| 400 | *c*_1_=0.3  *c*_2_=0.7 | 0.462 | 0.831 | 0.459 | 0.835 | 0.465 | 0.835 | 0.000 | 0.000 | 0.000 | 0.000 | 0.084 | 0.993 |
| 400 | *c*_1_=0.4  *c*_2_=0.8 | 0.847 | 0.914 | 0.844 | 0.917 | 0.846 | 0.915 | 0.000 | 0.001 | 0.001 | 0.002 | 0.416 | 0.997 |
| 600 | *c*_1_=0.2  *c*_2_=0.6 | 0.190 | 0.874 | 0.188 | 0.871 | 0.189 | 0.874 | 0.000 | 0.000 | 0.001 | 0.004 | 0.033 | 0.996 |
| 600 | *c*_1_=0.3  *c*_2_=0.7 | 0.717 | 0.944 | 0.714 | 0.944 | 0.717 | 0.945 | 0.000 | 0.000 | 0.005 | 0.012 | 0.308 | 0.997 |
| 600 | *c*_1_=0.4  *c*_2_=0.8 | 0.947 | 0.985 | 0.947 | 0.986 | 0.948 | 0.986 | 0.000 | 0.009 | 0.030 | 0.032 | 0.816 | 0.995 |

Table S9. Power comparison among methods under the linear CWSM model in Case II.

| *n* | *c* | NL-HSIM(PA) | | NL-HSIM(A) | | NL-HSIM(O) | | SKAT(L) | | SKAT(IBS) | | aSPU | |
| --- | --- | --- | --- | --- | --- | --- | --- | --- | --- | --- | --- | --- | --- |
|  |  | Group1 | Group2 | Group1 | Group2 | Group1 | Group2 | Group1 | Group2 | Group1 | Group2 | Group1 | Group2 |
| 400 | *c*_1_=0.2  *c*_2_=0.05 | 0.291 | 0.124 | 0.297 | 0.123 | 0.294 | 0.124 | 0.000 | 0.002 | 0.057 | 0.013 | 0.356 | 0.137 |
| 400 | *c*_1_=0.25  *c*_2_=0.075 | 0.621 | 0.765 | 0.622 | 0.765 | 0.621 | 0.765 | 0.000 | 0.027 | 0.250 | 0.247 | 0.778 | 0.833 |
| 400 | *c*_1_=0.3  *c*_2_=1 | 0.882 | 0.987 | 0.887 | 0.988 | 0.885 | 0.988 | 0.002 | 0.173 | 0.581 | 0.876 | 0.965 | 0.998 |
| 600 | *c*_1_=0.2  *c*_2_=0.05 | 0.594 | 0.360 | 0.597 | 0.358 | 0.604 | 0.360 | 0.000 | 0.008 | 0.279 | 0.108 | 0.796 | 0.484 |
| 600 | *c*_1_=0.25  *c*_2_=0.075 | 0.914 | 0.973 | 0.915 | 0.975 | 0.915 | 0.977 | 0.001 | 0.173 | 0.761 | 0.847 | 0.988 | 0.993 |
| 600 | *c*_1_=0.3  *c*_2_=1 | 0.995 | 1.000 | 0.994 | 1.000 | 0.995 | 1.000 | 0.005 | 0.665 | 0.973 | 1.000 | 1.000 | 1.000 |

Table S10. Power comparison among methods under the linear DSSM model in Case II.

| *n* | *c* | NL-HSIM(PA) | | NL-HSIM(A) | | NL-HSIM(O) | | SKAT(L) | | SKAT(IBS) | | aSPU | |
| --- | --- | --- | --- | --- | --- | --- | --- | --- | --- | --- | --- | --- | --- |
|  |  | Group1 | Group2 | Group1 | Group2 | Group1 | Group2 | Group1 | Group2 | Group1 | Group2 | Group1 | Group2 |
| 400 | *c*_1_=1  *c*_2_=1 | 0.463 | 0.623 | 0.456 | 0.622 | 0.460 | 0.621 | 0.001 | 0.000 | 0.003 | 0.000 | 0.680 | 0.983 |
| 400 | *c*_1_=1.5  *c*_2_=1.5 | 0.928 | 0.937 | 0.927 | 0.936 | 0.927 | 0.936 | 0.000 | 0.005 | 0.062 | 0.006 | 0.999 | 0.997 |
| 400 | *c*_1_=2  *c*_2_=2 | 0.997 | 0.987 | 0.996 | 0.988 | 0.997 | 0.987 | 0.000 | 0.029 | 0.435 | 0.036 | 1.000 | 0.999 |
| 600 | *c*_1_=1  *c*_2_=1 | 0.695 | 0.778 | 0.698 | 0.781 | 0.696 | 0.782 | 0.000 | 0.000 | 0.029 | 0.001 | 0.957 | 0.996 |
| 600 | *c*_1_=1.5  *c*_2_=1.5 | 0.984 | 0.993 | 0.985 | 0.993 | 0.983 | 0.994 | 0.000 | 0.034 | 0.395 | 0.08 | 1.000 | 0.997 |
| 600 | *c*_1_=2  *c*_2_=2 | 1.000 | 0.999 | 1.000 | 0.999 | 1.000 | 0.999 | 0.000 | 0.241 | 0.958 | 0.473 | 1.000 | 0.997 |

Table S11. Type I error comparison among methods in Case III.

| *n* | *ρ* | NL-HSIM(P) | | NL-HSIM(A) | | NL-HSIM(PA) | | NL-HSIM(O) | | Linear-HSIM | |
| --- | --- | --- | --- | --- | --- | --- | --- | --- | --- | --- | --- |
|  |  | Group1 | Group2 | Group1 | Group2 | Group1 | Group2 | Group1 | Group2 | Group1 | Group2 |
| 400 | 0.2 | 0.032 | 0.028 | 0.063 | 0.049 | 0.063 | 0.047 | 0.064 | 0.048 | 0.073 | 0.063 |
|  | 0.5 | 0.027 | 0.031 | 0.055 | 0.047 | 0.055 | 0.050 | 0.056 | 0.049 | 0.054 | 0.060 |
|  | 0.8 | 0.025 | 0.024 | 0.045 | 0.057 | 0.044 | 0.057 | 0.046 | 0.057 | 0.053 | 0.069 |
| 600 | 0.2 | 0.024 | 0.032 | 0.050 | 0.049 | 0.053 | 0.049 | 0.051 | 0.049 | 0.048 | 0.063 |
|  | 0.5 | 0.029 | 0.026 | 0.051 | 0.050 | 0.051 | 0.051 | 0.051 | 0.050 | 0.060 | 0.061 |
|  | 0.8 | 0.034 | 0.032 | 0.051 | 0.048 | 0.044 | 0.049 | 0.049 | 0.047 | 0.052 | 0.053 |

Table S12. Power comparison among methods under the nonlinear CWSM model in Case III.

| *c* | *n* | *ρ* | NL-HSIM(P) | | NL-HSIM(A) | | NL-HSIM(PA) | | NL-HSIM(O) | | Linear-HSIM | |
| --- | --- | --- | --- | --- | --- | --- | --- | --- | --- | --- | --- | --- |
|  |  |  | Group1 | Group2 | Group1 | Group2 | Group1 | Group2 | Group1 | Group2 | Group1 | Group2 |
| *c*_1_=0.06  *c*_2_=0.07 | 400 | 0.2 | 0.033 | 0.030 | 0.067 | 0.054 | 0.071 | 0.056 | 0.069 | 0.055 | 0.064 | 0.054 |
|  |  | 0.5 | 0.043 | 0.021 | 0.134 | 0.082 | 0.132 | 0.083 | 0.136 | 0.083 | 0.063 | 0.060 |
|  |  | 0.8 | 0.687 | 0.644 | 0.931 | 0.925 | 0.931 | 0.925 | 0.931 | 0.925 | 0.051 | 0.064 |
|  | 600 | 0.2 | 0.041 | 0.030 | 0.101 | 0.077 | 0.100 | 0.076 | 0.102 | 0.077 | 0.049 | 0.054 |
|  |  | 0.5 | 0.044 | 0.034 | 0.220 | 0.173 | 0.222 | 0.174 | 0.222 | 0.172 | 0.047 | 0.051 |
|  |  | 0.8 | 0.982 | 0.987 | 0.999 | 1.000 | 0.999 | 1.000 | 0.999 | 1.000 | 0.058 | 0.061 |
| *c*_1_=0.07  *c*_2_=0.08 | 400 | 0.2 | 0.034 | 0.027 | 0.081 | 0.079 | 0.081 | 0.076 | 0.080 | 0.078 | 0.063 | 0.053 |
|  |  | 0.5 | 0.048 | 0.037 | 0.180 | 0.119 | 0.183 | 0.117 | 0.183 | 0.118 | 0.058 | 0.058 |
|  |  | 0.8 | 0.864 | 0.750 | 0.978 | 0.966 | 0.980 | 0.966 | 0.980 | 0.965 | 0.062 | 0.050 |
|  | 600 | 0.2 | 0.047 | 0.032 | 0.126 | 0.088 | 0.130 | 0.090 | 0.131 | 0.088 | 0.053 | 0.055 |
|  |  | 0.5 | 0.109 | 0.038 | 0.365 | 0.247 | 0.365 | 0.247 | 0.364 | 0.247 | 0.047 | 0.056 |
|  |  | 0.8 | 0.999 | 0.997 | 1.000 | 1.000 | 1.000 | 1.000 | 1.000 | 1.000 | 0.055 | 0.051 |
| *c*_1_=0.08  *c*_2_=0.09 | 400 | 0.2 | 0.038 | 0.026 | 0.093 | 0.055 | 0.095 | 0.052 | 0.095 | 0.053 | 0.070 | 0.065 |
|  |  | 0.5 | 0.063 | 0.044 | 0.235 | 0.142 | 0.237 | 0.142 | 0.237 | 0.143 | 0.058 | 0.058 |
|  |  | 0.8 | 0.954 | 0.825 | 1.000 | 0.980 | 1.000 | 0.980 | 1.000 | 0.980 | 0.056 | 0.065 |
|  | 600 | 0.2 | 0.109 | 0.038 | 0.365 | 0.087 | 0.365 | 0.086 | 0.364 | 0.087 | 0.047 | 0.050 |
|  |  | 0.5 | 0.201 | 0.054 | 0.533 | 0.294 | 0.528 | 0.298 | 0.532 | 0.294 | 0.043 | 0.060 |
|  |  | 0.8 | 1.000 | 1.000 | 1.000 | 1.000 | 1.000 | 1.000 | 1.000 | 1.000 | 0.052 | 0.062 |

Table S13. Power comparison among methods under the nonlinear DSSM model in Case III.

| *c* | *n* | *ρ* | NL-HSIM(P) | | NL-HSIM(A) | | NL-HSIM(PA) | | NL-HSIM(O) | | Linear-HSIM | |
| --- | --- | --- | --- | --- | --- | --- | --- | --- | --- | --- | --- | --- |
|  |  |  | Group1 | Group2 | Group1 | Group2 | Group1 | Group2 | Group1 | Group2 | Group1 | Group2 |
| *c*_1_=0.3  *c*_2_=0.4 | 400 | 0.2 | 0.145 | 0.050 | 0.526 | 0.335 | 0.524 | 0.336 | 0.527 | 0.337 | 0.044 | 0.059 |
|  |  | 0.5 | 0.142 | 0.093 | 0.526 | 0.597 | 0.532 | 0.597 | 0.530 | 0.597 | 0.068 | 0.062 |
|  |  | 0.8 | 0.212 | 0.438 | 0.627 | 0.964 | 0.636 | 0.965 | 0.634 | 0.965 | 0.064 | 0.056 |
|  | 600 | 0.2 | 0.185 | 0.030 | 0.691 | 0.309 | 0.694 | 0.310 | 0.694 | 0.310 | 0.046 | 0.034 |
|  |  | 0.5 | 0.210 | 0.076 | 0.698 | 0.596 | 0.699 | 0.595 | 0.697 | 0.597 | 0.048 | 0.042 |
|  |  | 0.8 | 0.320 | 0.523 | 0.800 | 0.981 | 0.806 | 0.980 | 0.802 | 0.981 | 0.059 | 0.051 |
| *c*_1_=0.4  *c*_2_=1.2 | 400 | 0.2 | 0.293 | 0.098 | 0.773 | 0.680 | 0.769 | 0.681 | 0.770 | 0.683 | 0.046 | 0.041 |
|  |  | 0.5 | 0.314 | 0.172 | 0.794 | 0.867 | 0.795 | 0.865 | 0.794 | 0.865 | 0.062 | 0.042 |
|  |  | 0.8 | 0.451 | 0.737 | 0.877 | 0.996 | 0.879 | 0.996 | 0.880 | 0.997 | 0.060 | 0.045 |
|  | 600 | 0.2 | 0.357 | 0.092 | 0.868 | 0.637 | 0.869 | 0.638 | 0.869 | 0.638 | 0.048 | 0.049 |
|  |  | 0.5 | 0.378 | 0.166 | 0.884 | 0.843 | 0.883 | 0.841 | 0.884 | 0.843 | 0.051 | 0.035 |
|  |  | 0.8 | 0.560 | 0.810 | 0.951 | 1.000 | 0.952 | 1.000 | 0.951 | 1.000 | 0.058 | 0.033 |
| *c*_1_=0.5  *c*_2_=2 | 400 | 0.2 | 0.458 | 0.117 | 0.893 | 0.721 | 0.895 | 0.719 | 0.895 | 0.720 | 0.045 | 0.048 |
|  |  | 0.5 | 0.454 | 0.197 | 0.912 | 0.895 | 0.909 | 0.894 | 0.911 | 0.896 | 0.066 | 0.047 |
|  |  | 0.8 | 0.650 | 0.766 | 0.957 | 1.000 | 0.960 | 1.000 | 0.959 | 1.000 | 0.057 | 0.050 |
|  | 600 | 0.2 | 0.522 | 0.079 | 0.935 | 0.679 | 0.935 | 0.681 | 0.936 | 0.680 | 0.056 | 0.046 |
|  |  | 0.5 | 0.567 | 0.188 | 0.959 | 0.874 | 0.960 | 0.873 | 0.960 | 0.875 | 0.055 | 0.047 |
|  |  | 0.8 | 0.773 | 0.821 | 0.987 | 0.999 | 0.987 | 0.999 | 0.987 | 0.999 | 0.064 | 0.029 |

Table S14. Power comparison among methods under the linear CWSM model in Case III.

| *c* | *n* | *ρ* | NL-HSIM(P) | | NL-HSIM(A) | | NL-HSIM(PA) | | NL-HSIM(O) | | Linear-HSIM | |
| --- | --- | --- | --- | --- | --- | --- | --- | --- | --- | --- | --- | --- |
|  |  |  | Group1 | Group2 | Group1 | Group2 | Group1 | Group2 | Group1 | Group2 | Group1 | Group2 |
| *c*_1_=0.03  *c*_2_=0.03 | 400 | 0.2 | 0.033 | 0.033 | 0.060 | 0.075 | 0.061 | 0.076 | 0.062 | 0.076 | 0.078 | 0.066 |
|  |  | 0.5 | 0.040 | 0.056 | 0.103 | 0.198 | 0.102 | 0.198 | 0.105 | 0.198 | 0.189 | 0.221 |
|  |  | 0.8 | 0.353 | 0.939 | 0.648 | 0.998 | 0.652 | 0.998 | 0.652 | 0.998 | 0.873 | 1.000 |
|  | 600 | 0.2 | 0.037 | 0.032 | 0.052 | 0.085 | 0.053 | 0.084 | 0.052 | 0.084 | 0.084 | 0.084 |
|  |  | 0.5 | 0.043 | 0.095 | 0.159 | 0.386 | 0.159 | 0.388 | 0.159 | 0.388 | 0.323 | 0.467 |
|  |  | 0.8 | 0.656 | 1.000 | 0.881 | 1.000 | 0.882 | 1.000 | 0.883 | 1.000 | 0.983 | 1.000 |
| *c*_1_=0.04  *c*_2_=0.04 | 400 | 0.2 | 0.027 | 0.040 | 0.064 | 0.084 | 0.066 | 0.083 | 0.064 | 0.084 | 0.102 | 0.105 |
|  |  | 0.5 | 0.068 | 0.132 | 0.205 | 0.419 | 0.205 | 0.418 | 0.206 | 0.419 | 0.374 | 0.563 |
|  |  | 0.8 | 0.767 | 0.999 | 0.939 | 1.000 | 0.940 | 1.000 | 0.940 | 1.000 | 0.992 | 1.000 |
|  | 600 | 0.2 | 0.033 | 0.040 | 0.089 | 0.127 | 0.088 | 0.127 | 0.089 | 0.126 | 0.139 | 0.151 |
|  |  | 0.5 | 0.137 | 0.366 | 0.407 | 0.816 | 0.403 | 0.820 | 0.407 | 0.816 | 0.662 | 0.892 |
|  |  | 0.8 | 0.961 | 1.000 | 0.993 | 1.000 | 0.993 | 1.000 | 0.993 | 1.000 | 0.999 | 1.000 |
| *c*_1_=0.05  *c*_2_=0.05 | 400 | 0.2 | 0.034 | 0.026 | 0.085 | 0.105 | 0.085 | 0.105 | 0.085 | 0.105 | 0.155 | 0.155 |
|  |  | 0.5 | 0.150 | 0.294 | 0.410 | 0.762 | 0.412 | 0.761 | 0.411 | 0.763 | 0.661 | 0.838 |
|  |  | 0.8 | 0.966 | 1.000 | 0.995 | 1.000 | 0.995 | 1.000 | 0.994 | 1.000 | 1.000 | 1.000 |
|  | 600 | 0.2 | 0.044 | 0.039 | 0.142 | 0.218 | 0.144 | 0.216 | 0.144 | 0.217 | 0.278 | 0.277 |
|  |  | 0.5 | 0.425 | 0.746 | 0.753 | 0.984 | 0.755 | 0.984 | 0.755 | 0.984 | 0.915 | 0.997 |
|  |  | 0.8 | 0.997 | 1.000 | 1.000 | 1.000 | 1.000 | 1.000 | 1.000 | 1.000 | 1.000 | 1.000 |

Table S15. Power comparison among methods under the linear DSSM model in Case III.

| *c* | *n* | *ρ* | NL-HSIM(P) | | NL-HSIM(A) | | NL-HSIM(PA) | | NL-HSIM(O) | | Linear-HSIM | |
| --- | --- | --- | --- | --- | --- | --- | --- | --- | --- | --- | --- | --- |
|  |  |  | Group1 | Group2 | Group1 | Group2 | Group1 | Group2 | Group1 | Group2 | Group1 | Group2 |
| *c*_1_=0.1  *c*_2_=0.1 | 400 | 0.2 | 0.030 | 0.024 | 0.076 | 0.054 | 0.076 | 0.054 | 0.077 | 0.054 | 0.099 | 0.064 |
|  |  | 0.5 | 0.032 | 0.025 | 0.059 | 0.062 | 0.060 | 0.063 | 0.058 | 0.063 | 0.077 | 0.070 |
|  |  | 0.8 | 0.021 | 0.036 | 0.062 | 0.062 | 0.063 | 0.060 | 0.062 | 0.061 | 0.095 | 0.054 |
|  | 600 | 0.2 | 0.029 | 0.022 | 0.062 | 0.063 | 0.062 | 0.063 | 0.063 | 0.063 | 0.108 | 0.061 |
|  |  | 0.5 | 0.024 | 0.029 | 0.063 | 0.064 | 0.065 | 0.065 | 0.062 | 0.065 | 0.101 | 0.072 |
|  |  | 0.8 | 0.041 | 0.028 | 0.068 | 0.080 | 0.072 | 0.081 | 0.073 | 0.081 | 0.123 | 0.076 |
| *c*_1_=0.3  *c*_2_=0.3 | 400 | 0.2 | 0.232 | 0.042 | 0.594 | 0.300 | 0.595 | 0.300 | 0.597 | 0.301 | 0.833 | 0.391 |
|  |  | 0.5 | 0.230 | 0.060 | 0.608 | 0.320 | 0.607 | 0.320 | 0.605 | 0.320 | 0.845 | 0.417 |
|  |  | 0.8 | 0.245 | 0.122 | 0.594 | 0.426 | 0.605 | 0.425 | 0.600 | 0.425 | 0.870 | 0.570 |
|  | 600 | 0.2 | 0.317 | 0.062 | 0.749 | 0.399 | 0.747 | 0.396 | 0.747 | 0.397 | 0.941 | 0.545 |
|  |  | 0.5 | 0.321 | 0.084 | 0.765 | 0.464 | 0.764 | 0.465 | 0.766 | 0.467 | 0.943 | 0.590 |
|  |  | 0.8 | 0.394 | 0.202 | 0.781 | 0.631 | 0.781 | 0.632 | 0.781 | 0.629 | 0.958 | 0.764 |
| *c*_1_=0.5  *c*_2_=0.5 | 400 | 0.2 | 0.773 | 0.184 | 0.982 | 0.753 | 0.982 | 0.753 | 0.982 | 0.754 | 1.000 | 0.920 |
|  |  | 0.5 | 0.789 | 0.316 | 0.985 | 0.823 | 0.985 | 0.825 | 0.985 | 0.824 | 1.000 | 0.932 |
|  |  | 0.8 | 0.764 | 0.603 | 0.983 | 0.933 | 0.981 | 0.932 | 0.983 | 0.933 | 0.998 | 0.982 |
|  | 600 | 0.2 | 0.902 | 0.231 | 0.999 | 0.869 | 1.000 | 0.870 | 0.999 | 0.870 | 1.000 | 0.971 |
|  |  | 0.5 | 0.919 | 0.468 | 0.999 | 0.937 | 0.999 | 0.935 | 0.999 | 0.936 | 1.000 | 0.985 |
|  |  | 0.8 | 0.892 | 0.791 | 0.996 | 0.986 | 0.995 | 0.986 | 0.995 | 0.986 | 1.000 | 0.998 |

Table S16. Properties of the cohort used for method validation.

| Covariates | Statistical Description |
| --- | --- |
| Age(years;mean±SD) | 74.6±6.8 |
| Gender(male/female(%)) | 34.5/65.5(%) |
| Education(Year;min/median(IQR)/max) | 6/16(4)/20 |
| APOE4(copy number,1/2/3(%)) | 50.7/37.9/11.4(%) |

| Brain region | Covariates / Gene name | *p*-value |
| --- | --- | --- |
| entorhinal cortex | *APOE4* | 2.153e-05 |
| entorhinal cortex | *AGE* | 3.222e-06 |
| entorhinal cortex | *GENDER* | 3.265e-05 |
| entorhinal cortex | *EDUCAT* | 6.746e-07 |
| ventricles | *AGE* | 1.925e-13 |
| ventricles | *GENDER* | 8.572e-18 |
| ventricles | *GPRC6A* | 0.027 |
| ventricles | *TCP10L2* | 0.023 |
| fusiform gyrus | *AGE* | 1.753e-05 |
| fusiform gyrus | *GENDER* | 1.230e-13 |
| fusiform gyrus | *EDUCAT* | 8.703e-06 |
| fusiform gyrus | *GALNT7* | <0.001 |
| fusiform gyrus | *KCNJ6* | 0.031 |
| fusiform gyrus | *ADORA2A-AS1, UPB1, SPECC1L-ADORA2A, ADORA2A* | 0.009 |
| hippocampus | *APOE4* | 7.992e-07 |
| hippocampus | *AGE* | 4.771e-14 |
| hippocampus | *GENDER* | 3.540e-03 |
| hippocampus | *CACNA1C, CACNA1C-IT1, CACNA1C-AS4，*  *CACNA1C-AS1，DCP1B* | <0.001 |
| middle temporal gyrus | *APOE4* | 0.0081 |
| middle temporal gyrus | *AGE* | 7.943e-05 |
| middle temporal gyrus | *GENDER* | 2.331e-12 |
| middle temporal gyrus | *MICAL3* | <0.001 |

Table S17. Significant associations between brain regions, covariates, and gene markers in the real data analysis.

**References**

1. Székely GJ, Rizzo ML, Bakirov NK. Measuring and testing dependence by correlation of distances, The Annals of Statistics 2007;35:2769–2794.<https://doi.org/10.1214/009053607000000505>.

2. Zhang K, Tsang IW, Kwok JT. Improved Nyström low-rank approximation and error analysis, Proceedings of the 25th international conference on Machine learning 2008:1232–1239.<https://doi.org/10.1145/1390156.1390311>.

3. Mika S, Schölkopf B, Smola A et al. Kernel PCA and de-noising in feature spaces, Advances in neural information processing systems 1998;11.

4. Wang Q. Kernel principal component analysis and its applications in face recognition and active shape models, arXiv preprint arXiv:1207.3538 2012.<https://doi.org/10.48550/arXiv.1207.3538>.

5. Zhang C-H, Zhang SS. Confidence intervals for low dimensional parameters in high dimensional linear models, Journal of the Royal Statistical Society Series B: Statistical Methodology 2014;76:217–242.<https://doi.org/10.1111/rssb.12026>.

6. Van de Geer S, Bühlmann P, Ritov Ya et al. On asymptotically optimal confidence regions and tests for high-dimensional models 2014.<https://doi.org/10.1214/14-AOS1221>.

7. Javanmard A, Montanari A. Confidence intervals and hypothesis testing for high-dimensional regression, The Journal of Machine Learning Research 2014;15:2869–2909.<https://doi.org/10.48550/arXiv.1306.3171>.
